# Computational identification of long non-coding RNAs associated with graphene therapy in glioblastoma multiforme

**DOI:** 10.1093/braincomms/fcad293

**Published:** 2023-10-25

**Authors:** Zhuoheng Zou, Ming Zhang, Shang Xu, Youzhong Zhang, Junzheng Zhang, Zesong Li, Xiao Zhu

**Affiliations:** Computational Systems Biology Lab (CSBL), The Marine Biomedical Research Institute, Guangdong Medical University, Zhanjiang 524023, China; Department of Physical Medicine and Rehabilitation, Zibo Central Hospital, Zibo 255000, China; Computational Systems Biology Lab (CSBL), The Marine Biomedical Research Institute, Guangdong Medical University, Zhanjiang 524023, China; Computational Systems Biology Lab (CSBL), The Marine Biomedical Research Institute, Guangdong Medical University, Zhanjiang 524023, China; Computational Systems Biology Lab (CSBL), The Marine Biomedical Research Institute, Guangdong Medical University, Zhanjiang 524023, China; Guangdong Provincial Key Laboratory of Systems Biology and Synthetic Biology for Urogenital Tumors, Shenzhen Key Laboratory of Genitourinary Tumor, Department of Urology, The First Affiliated Hospital of Shenzhen University, Shenzhen Second People’s Hospital (Shenzhen Institute of Translational Medicine), Shenzhen 518035, China; Computational Systems Biology Lab (CSBL), The Marine Biomedical Research Institute, Guangdong Medical University, Zhanjiang 524023, China

**Keywords:** graphene oxide therapy, lncRNAs, independent prognostic model, glioblastoma multiforme, drug screening

## Abstract

Glioblastoma multiforme represents the most prevalent primary malignant brain tumour, while long non-coding RNA assumes a pivotal role in the pathogenesis and progression of glioblastoma multiforme. Nonetheless, the successful delivery of long non-coding RNA-based therapeutics to the tumour site has encountered significant obstacles attributable to inadequate biocompatibility and inefficient drug delivery systems. In this context, the use of a biofunctional surface modification of graphene oxide has emerged as a promising strategy to surmount these challenges. By changing the surface of graphene oxide, enhanced biocompatibility can be achieved, facilitating efficient transport of long non-coding RNA-based therapeutics specifically to the tumour site. This innovative approach presents the opportunity to exploit the therapeutic potential inherent in long non-coding RNA biology for treating glioblastoma multiforme patients. This study aimed to extract relevant genes from The Cancer Genome Atlas database and associate them with long non-coding RNAs to identify graphene therapy–related long non-coding RNA. We conducted a series of analyses to achieve this goal, including univariate Cox regression, least absolute shrinkage and selection operator regression and multivariate Cox regression. The resulting graphene therapy–related long non-coding RNAs were utilized to develop a risk score model. Subsequently, we conducted Gene Ontology and Kyoto Encyclopedia of Genes and Genomes pathway analyses on the identified graphene therapy–related long non-coding RNAs. Additionally, we employed the risk model to construct the tumour microenvironment model and analyse drug sensitivity. To validate our findings, we referenced the IMvigor210 immunotherapy model. Finally, we investigated differences in the tumour stemness index. Through our investigation, we identified four promising graphene therapy–related long non-coding RNAs (AC011405.1, HOXC13-AS, LINC01127 and LINC01574) that could be utilized for treating glioblastoma multiforme patients. Furthermore, we identified 16 compounds that could be utilized in graphene therapy. Our study offers novel insights into the treatment of glioblastoma multiforme, and the identified graphene therapy–related long non-coding RNAs and compounds hold promise for further research in this field. Furthermore, additional biological experiments will be essential to validate the clinical significance of our model. These experiments can help confirm the potential therapeutic value and efficacy of the identified graphene therapy–related long non-coding RNAs and compounds in treating glioblastoma multiforme.

## Teaser

Zou *et al*. identified four graphene therapy–related long non-coding RNAs as prognostic markers for glioblastoma multiforme. In addition, they investigated drug sensitivity and immune pathways to develop potential treatments utilizing graphene oxide nanocarriers.

## Graphical Abstract

**Graphical Abstract:**
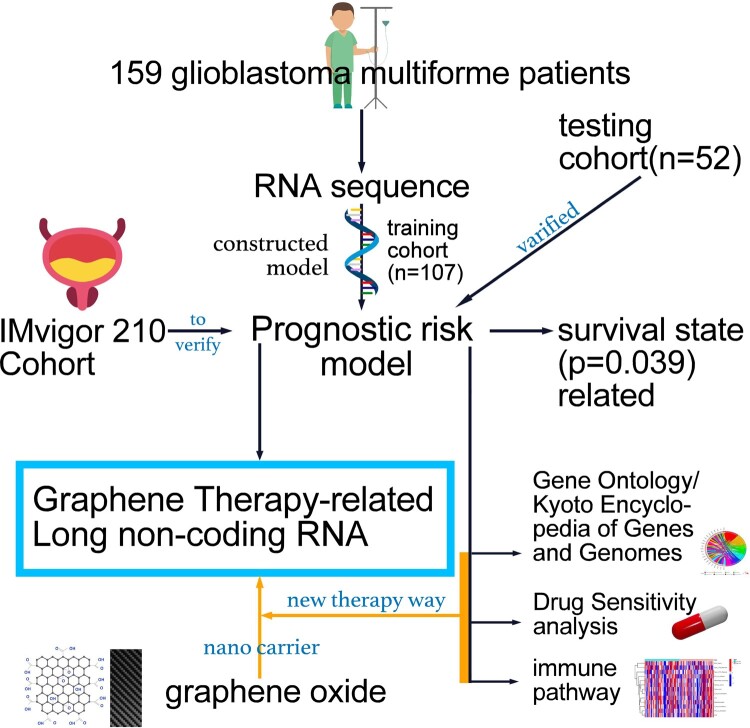

## Introduction

Graphene, a class of 2D sp2 carbon nanomaterials, holds great potential for biomedical applications, including anti-tumour therapy. The 2D structure of graphene nanosheets provides ample active sites and enough space to interact with functional groups through conjugation reactions or absorption by various therapeutic modalities.^1^ Both graphene oxide and reduced graphene oxide possess plasmonic properties that can convert laser energy into heat.^2^  *In vitro* studies have demonstrated that sandwich-like graphene oxide nanosheets significantly enhance drug penetration and internalization, followed by near-infrared–controlled endo/lysosomal disruption for tumor-targeted multimodal doxorubicin (iAPG) release [iAPG: internalizing RGD (Arg-Gly-Asp) peptide conjugated apoA-I protein graphene oxide]. Furthermore, iAPG-mediated site-specific drug shuttling increases tumour drug accumulation by 3.53-fold compared with free doxorubicin (DOX) *in vivo* and significantly induces deep tumour penetration. Under optimal near-infrared conditions, primary tumour ablation and spontaneous metastasis inhibition were further demonstrated with negligible side effects.^3^ Another study investigated the tissue distribution and anticancer effects of graphene oxide conjugated with paclitaxel. Graphene oxide–conjugated paclitaxel showed good solubility and biocompatibility.^4^ In addition, compared with free paclitaxel, the conjugated form demonstrated longer circulation time, higher tumour targeting and stronger tumour suppressive effects.^5^ These findings suggest the potential of graphene-based therapies in improving drug delivery and enhancing treatment efficacy in cancer.

Gliomas are the most prevalent primary intracranial tumours, comprising 81% of malignant brain tumours. Among glioma histologies, glioblastoma multiforme (GBM) accounts for 45% of all gliomas and has a 5-year relative disease course survival rate of 5%.^6^ Despite improving treatment options, GBM remains the most challenging type of cancer to manage, requiring a multidisciplinary understanding and approach to the disease and its potential complications.^7^ The publication of the fifth edition of the World Health Organization Classification of Tumours of the Central Nervous System (WHO CNS-5) in 2021 emphasizes the significance of molecular diagnostics in classifying CNS tumours.^8^ The survival rate of GBM patients is influenced by a single molecular biomarker.^9^ Therefore, studying the molecular variability of GBMs and their impact on individual GBMs’ biological behaviour is crucial for making an accurate diagnosis, prognosis and prediction of treatment response.

Long non-coding RNAs (lncRNAs) are RNA molecules with a conserved secondary structure that do not encode proteins and are longer than 200 nucleotides.^10^ These molecules can be found in body fluids such as serum, plasma and urine and can be cancer detection and prognostic biomarkers for various cancers.^11^ In GBM, there is ongoing research investigating lncRNAs, to identify lncRNAs that can serve as diagnostic, prognostic or predictive markers and correlate with tumour progression and survival.^12^ The role of lncRNAs in GBM is becoming an area of intense research. For example, ASLNC22381 and ASLNC081 may be involved in recurrence and progression, highlighting the potential of using lncRNA and mRNA analysis to detect GBM.^13^

GBM is a challenging type of cancer, and effective treatment options are still non-existent. lncRNAs have been shown to influence various aspects of tumour development, metastasis, recurrence, invasiveness and drug sensitivity. Meanwhile, graphene oxide has shown great promise in targeting tumours due to its unique properties. However, research on GBM treated with graphene oxide is non-existent. In this study, we identified that lncRNAs are associated with the overall survival (OS) of GBM patients and validated their reliability and sensitivity. We also investigated this signalling model’s immune regulation and drug sensitivity and performed enrichment analysis to gain insight into potential regulatory mechanisms. Our findings suggest that combining these identified lncRNAs with graphene oxide treatment could provide a novel diagnostic and therapeutic approach for GBM patients.

## Materials and methods

### Data resource

Graphene therapy–related genes are obtained from the Rat Genome Database, which contains genetic, genomic, phenotypic and disease-related data for multiple species, including humans and rats (https://rgd.mcw.edu/rgdweb/homepage/).^14^ A total of 220 genes were identified for our study. We obtained transcriptome sequencing data and clinical data from The Cancer Genome Atlas-GBM of the GDC database (https://portal.gdc.cancer.gov/) and retained information such as survival time, survival status, age and gender for patients.^15^ The genders of the individuals in the study are shown in Supplementary Table 1. Data from the IMvigor210 clinical trial were obtained from the European Genome-Phenome Archive. We used the ‘limma’ package and R software (version 4.0.2; https://cran.r-project.org/) to determine the co-expression relationship between gene sets and lncRNAs and displayed the results using a Sankey diagram.

### Building a graphene therapy–related lncRNA risk score model

One hundred and fifty-nine GBM patients were randomly assigned to training (*n* = 107) and testing cohorts (*n* = 52). First, we extracted the expression matrix of lncRNAs co-expressed with the graphene therapy–related genes. Using univariate Cox regression analysis, we identified 221 graphene therapy–related lncRNAs (GTLncRNAs) associated with prognosis. Then, the least absolute shrinkage and selection operator (LASSO) and multivariate Cox regressions were used to narrow the number of GTLncRNAs. Next, we calculated the risk score of the GTLncRNA signature of each patient using the following formula:


$$\mathrm{GTLncSigs}\;=\overset{n}{\underset{i=1}{\sum }}\;\mathrm{Coef}(i)\times x(i)$$

Coef(i) is the multivariate Cox regression coefficients corresponding to genes and x(i) the expression values of GTLncRNAs.^16^

We then divided patients into low-risk and high-risk groups based on the median of the risk scores by Kaplan–Meier survival curve analysis. Finally, the training cohort was used to build the predictive GTLncRNA-based gene signature (GTLncSig) model, and the entire cohort and test cohort were used to validate it.

### Prognostic model construction combined with clinical information

First, we assessed the risk scores of clinical features, including survival state, gender and age. Then, using the ‘ggpubr’ package, we created boxplots to identify outliers and observe the overall risk score distribution. Subsequently, we conducted univariate independent prognostic analysis and established a clinical multivariate Cox regression model. This analysis aimed to determine clinical factors that independently predict patient prognosis.

### Quality assessment of GTLncSig clinical prognostic model and verification of nomogram

The training set was used to construct a prognostic model, while the testing set was utilized to validate the model’s accuracy. We utilized ‘dplyr’, ‘survival’, ‘rms’ and ‘pec’ of the R package to construct the *C*-index for analysing the independent prognostic model of GTLncSig. The receiver operating characteristic (ROC) curve was constructed using the ‘survival’, ‘survminer’ and ‘time ROC’ packages of R.^17^ Furthermore, we investigated independent predictors associated with various clinical characteristics, including age, sex, risk score and survival state. We also assessed the specificity and sensitivity of the predictive signal for 1-, 3- and 5-year OS in GBM patients. We constructed a nomogram to predict 1-, 3- and 5-year OS based on different clinical features such as age, gender and risk scores.^18-20^ To assess the performance of this risk model, we constructed calibration curves using ‘survival’, ‘regplot’ and ‘RMS’ of the R packages.

### Analysis of discrimination by 3D principal component analysis

We used principal component analysis (PCA) for dimensionality reduction, model identification and visualization of expression data of lncRNAs, mRNAs and risk lncRNAs related to graphene therapy and prognosis in GBM patients. We used ‘scatterplot3d’ of the R package to cluster the expression data of lncRNAs, mRNAs and risk lncRNAs in GBM patients, and a 3D scatter plot was used to show the distribution of GBM patients.^21^

### Gene Ontology/Kyoto Encyclopedia of Genes and Genomes enrichment analysis

To perform Gene Ontology (GO)/Kyoto Encyclopedia of Genes and Genomes (KEGG) enrichment analysis of the risk difference data, we installed the R packages ‘Colorspace’, ‘stringi’, ‘dplyr’, ‘GGplot2’, ‘GGpubr’ and ‘BiocManager’. Gene names were converted to gene IDs, and GO/KEGG enrichment analysis was conducted to identify GTLncRNAs and distinguish them from unrelated lncRNAs. The enrichment analysis results for molecular function, biological process (BP), and cellular component were plotted as histograms.

### Study on the immune function of GTLncSig high-risk and low-risk groups

First, we installed ‘limma’, ‘GSVA’, ‘GSEABase’, ‘heatmap’ and ‘reshape2’ packages of R software and read the input files: gene expression data, immune function gene set and risk file.^22^ Then, we performed gene set variation analysis to estimate changes in the pathway and BP activity in the sample population without supervision.^23^ We also conducted a tumour mutation burden (TMB) analysis on samples from the high-risk and low-risk groups and generated a heatmap to visualize the results. TMB is defined as the number of somatic mutations per megabase of genomic sequence interrogated and varies by malignancy tumours.^24,25^ We used the tumour immune dysfunction and escape (TIDE) score (http://tide.dfci.harvard.edu/) to evaluate immune escape and immunotherapy effects in the high-risk and low-risk groups of GBM patients. We visualized the results using violin plots, where a higher TIDE score indicates a greater possibility of immune escape and a worse response to immunotherapy. In addition, we analysed other immune system biomarkers or cells, including the microsatellite instability (MSI) score,^26^ Merck18, a cluster of differentiation 274 (CD274),^27^ IFGN, CD8,^28,29^ myeloid-derived suppressor cell (MDSC),^30^ cancer-associated fibroblasts (CAFs)^31^ and tumour-associated macrophages M2 (TAM.M2).^32^

### Drug resistance exploration and screening

To investigate potential drugs for treating GBM patients using our GTLncRNA model, we installed the ‘GGpubr’, ‘pRRophetic’, and ‘ggplot2’ packages of R software and prepared risk files and expression data files for all samples.^33^ The data of all samples were read using expression and analysed to obtain semi-inhibitory concentrations (IC50) based on Genomics of Drug Sensitivity in Cancer, which were used to estimate the treatment response of the samples and identify potential drugs for GBM patients based on our GTLncRNA model.^34^

### IMvigor210 cohort evaluation of immunotherapy

To assess the potential of the GTLncRNA model in predicting immunotherapy response, we validated it using the IMvigor210 model. The IMvigor210 study evaluates the efficacy and safety of programmed cell death platinum-treated ligand 1 targeting antibody atezolizumab in patients with locally advanced or metastatic urothelial disease.^35^ First, we used the IMvigor210CoreBiologies software package to download the data and matched the genes obtained from LASSO regression analysis with the IMvigor210 model. Then, using the same formula, we calculated the risk scores for each item in the IMvigor210 cohort and divided the patients into high-risk and low-risk subgroups.

### Calculation and analysis of stemness indices (mRNAsi)

Stemness characteristics were obtained from the Progenitor Cell Biology Consortium database and identified using the one-class logistic regression algorithm.^36^ The degree of tumour de-differentiation can be evaluated using the mRNA Signal Intensity Index (mRNAsi) value, which corresponds to the level of stemness, with higher values indicating a more malignant tumour.^25^ Using RNA-seq data from The Cancer Genome Atlas, we conducted a cohort study of the stem cell index (mRNAsi) per patient based on the one-class logistic regression algorithm. The entire research process is shown in Supplementary Fig. 1.

### Statistical analyses

Perl programming is used for data processing. R software (4.1.2) was used for statistical analysis. Survival analysis was performed using the Kaplan–Meier curve. A prediction model was constructed through univariate Cox regression and LASSO regression, where a significance level of *P* < 0.05 was considered statistically significant.

### Ethical approval and consent to participate

The work was approved by the Guangdong Medical University committee (YS2021159 and YS2021265). Informed consent forms are not required for patient data extracted from public databases. As a cohort study, we adhered to the STrengthening the Reporting of OBservational studies in Epidemiology (STROBE) checklist.

## Results

### Identification of GTLncRNAs

We used the ‘limma’ package of R to perform differential analysis and identified 2088 lncRNAs significantly correlated with 198 associated genes. The results were visualized by the Sankey diagram (Supplementary Fig. 2). The univariate Cox regression analysis revealed 221 GTLncRNAs, which had a significant difference (*P* < 0.05), and the expression of ZMIZ1-AS1, AC131009.1, AC005264.1, LINC01943 and LINC01574 was substantial, with *P*-values <0.0003 (Supplementary Table 2).

### Discovery of four GTLncRNAs in a training cohort of patients with GBM

**Figure 1:**
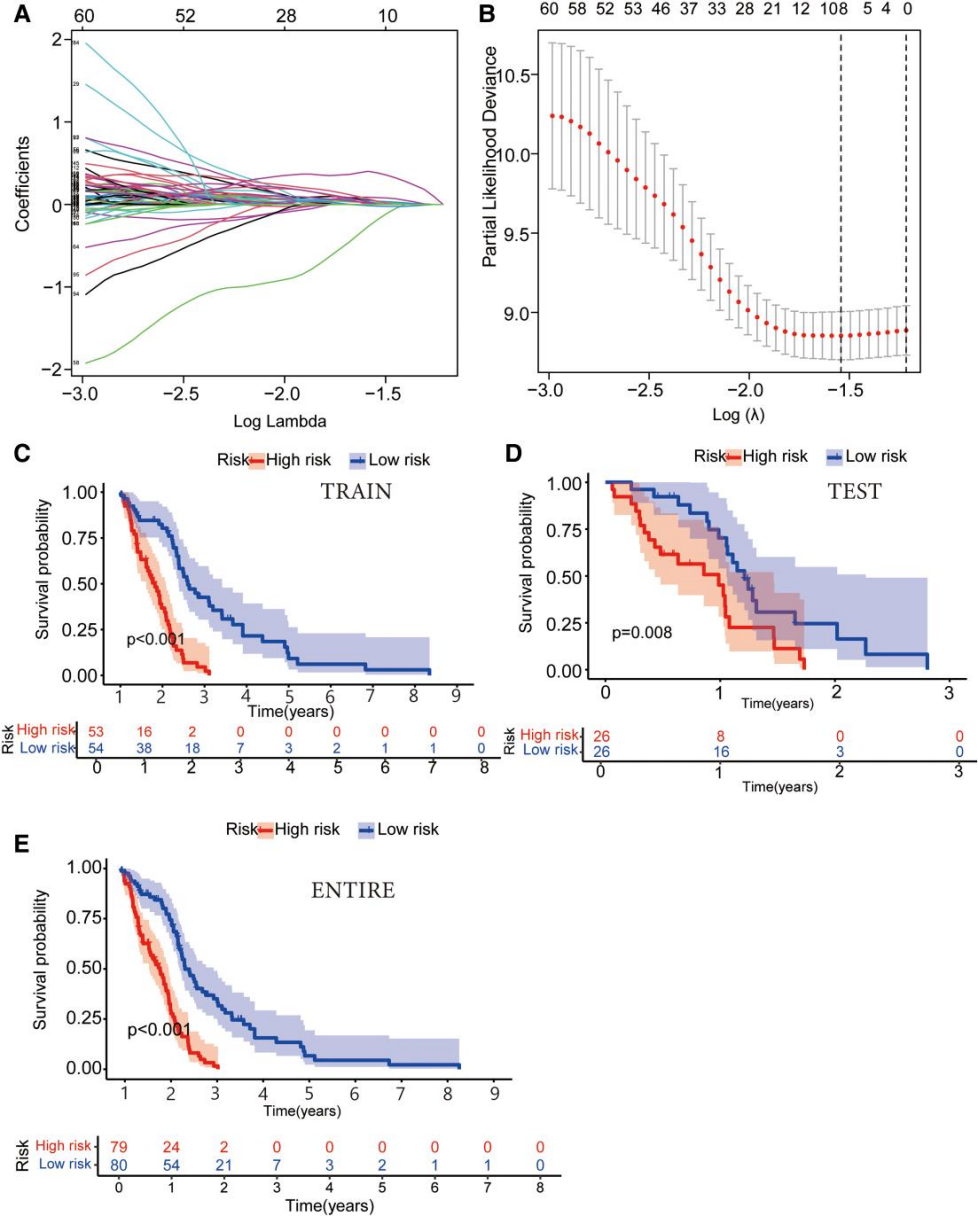
**Identification of target genes using LASSO Cox regression analysis and assessment of survival probability in different risk groups of GBM patients.** (**A**) Cross-verification of error curve via tuning parameters of OS-related proteins. This segment involves the utilization of tuning parameters associated with OS-related proteins. The objective is to cross-verify the error curve, thereby ensuring the robustness and reliability of the analysis. (**B**) Calculation of minimum criteria using perpendicular imaginary lines: a LASSO Cox regression analysis employing perpendicular imaginary lines is applied to establish the minimum criteria, which aids in accurately determining the crucial thresholds and boundary points for further research. (**C–E**) The Kaplan–Meier survival curve analysis illustrated differences in the survival probability of GBM patients of different risk groups in training (*n* = 107; *P* < 0.001), testing (*n* = 51; *P* = 0.008) and entire groups (*n* = 159; *P* < 0.001). The function calculates and displays the log-rank test *P*-value for the survival difference between the risk groups.

We randomly divided GBM patients (*n* = 159) into the training cohort (*n* = 107) and the testing cohort (*n* = 52) and guaranteed no significant difference in clinical characteristics (*P* > 0.05). Next, we analysed the risk characteristics for prognosis using lncRNA gene expression data in the training cohort. A prognostic model was constructed using LASSO Cox regression analysis (Fig. 1A and B). The multivariate Cox regression analysis identified four GTLncRNAs, AC011405.1, HOXC13-AS, LINC01127 and LINC01574, which could be potential targets for graphene oxide therapy (*P* < 0.05; Supplementary Table 3). Besides, we analysed the expression correlation between the four GTLncRNAs and the related genes. According to the result, the expressions of LINC01127 and LINC01574 were positively related to most genes (Supplementary Fig. 3).

### GTLncSig risk model function

Patients were divided into the high-risk (*n* = 53) and low-risk (*n* = 54) subgroups based on risk scores. The median risk score was used as the threshold to divide the patients. The Kaplan–Meier survival curve analysis showed that the OS time of GBM patients in the high-risk subgroup was significantly lower than that in the low-risk GBM subgroup (*P* < 0.001; Fig. 1C–E). The risk score distribution of patients in different subgroups illustrated that the high-risk subgroup had significantly lower survival than the low-risk subgroup (Supplementary Fig. 4A and B). The heat map showed that HOXC13-AS, LINC01127 and LINC01574 were highly expressed in the high-risk group, while their expression was lower in the low-risk group. On the other hand, the AC011405.1 expression was higher in the low-risk group (Supplementary Fig. 4C). Finally, we verified the signal of the test group model, and the resulting trend was consistent with the training group (Supplementary Fig. 4D–F).

### Relationship between GTLncSig risk scores and clinicopathological parameters of GBM patients

We visualized the relationship between clinicopathological factors and GTLncSig risk scores (Supplementary Fig. 4G). As can be seen, age (*P* = 0.13), gender (*P* = 0.21) and risk score were not correlated (*P* > 0.05), while survival state (*P* = 0.0039) was correlated with risk score (*P* < 0.05).

### Clinical independent prognostic value of GTLncSig prognostic model

**Figure 2:**
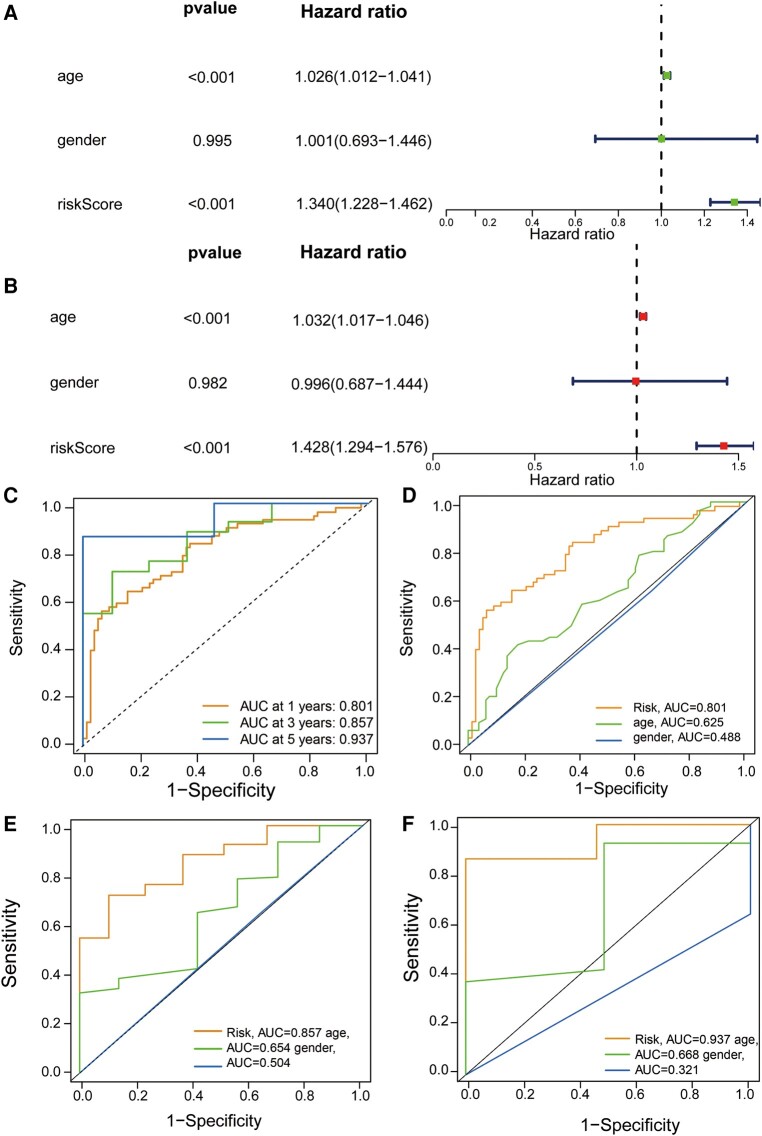
**Assessment of GTLncSig risk model’s independent prognostic value and performance and ROC curves.** (**A**) Exploration through univariate Cox regression analysis encompasses an in-depth exploration via univariate Cox regression analysis and systematically examines the individual impact of diverse factors (age and risk score, *P* < 0.001) on survival outcomes, enabling the identification of noteworthy contributors to patient prognosis. (**B**) The multivariate Cox regression analysis shows the same result as the univariate Cox regression analysis (age and risk score, *P* < 0.001). It identifies the clinical variables significantly affecting patient survival using univariate and multivariate Cox proportional hazards models. It visually represents hazard ratios, confidence intervals and *P*-values, aiding in the interpretation of variable significance. (**C**) The receiver operating characteristic curve of the optimal prognostic model (*n* = 594) for 1, 3 and 5 years shows AUC values of 0.801, 0.857 and 0.937, respectively. It is used to assess the quality of patients’ clinical independence, with over 0.5 being good. Risk scores are generally better than other factors as predictors, and other factors can be used as reference factors. The comparison of the receiver operating characteristic curves at 1 (**D**), 3 (**E**) and 5 years (**F**) with clinical features (risk *n* = 159; age and gender *n* = 594) are shown.

We performed univariate and multivariate independent predictive analyses to identify clinicopathological parameters such as age, gender and risk scores to determine whether the model was valid independently of other clinical characteristics. In the entire cohort, age and risk scores were significantly correlated with OS according to the univariate Cox regression analysis (Fig. 2A). In the multivariate Cox regression analysis, the results we obtained were also consistent with that in the univariate Cox regression analysis (Fig. 2B), suggesting that these two factors can function as independent prognostic factors independently of other clinical features. Next, we assessed the quality of patients’ clinical independence using a prognostic model of the ROC curve. According to the results shown, the areas under the curve (AUCs) of 1, 3 and 5 years are highly >0.5 (1-year AUC = 0.801, 3-year AUC = 0.857, 5-year AUC = 0.937, Fig. 2C). In the decisive 1-year survival of GBM patients, risk scores (AUC = 0.801) and age (AUC = 0.625) were significant, while gender (AUC = 0.488) was insignificant (Fig. 2D). We illustrate the 3- and 5-year survival rates, which almost remained the same as the 1-year survival rate in Fig. 2E and F. The risk score was generally better than other factors to be a predictor, and other factors can be treated as reference factors.

### Nomograms and signal model validation for clinical subgroup data

**Figure 3:**
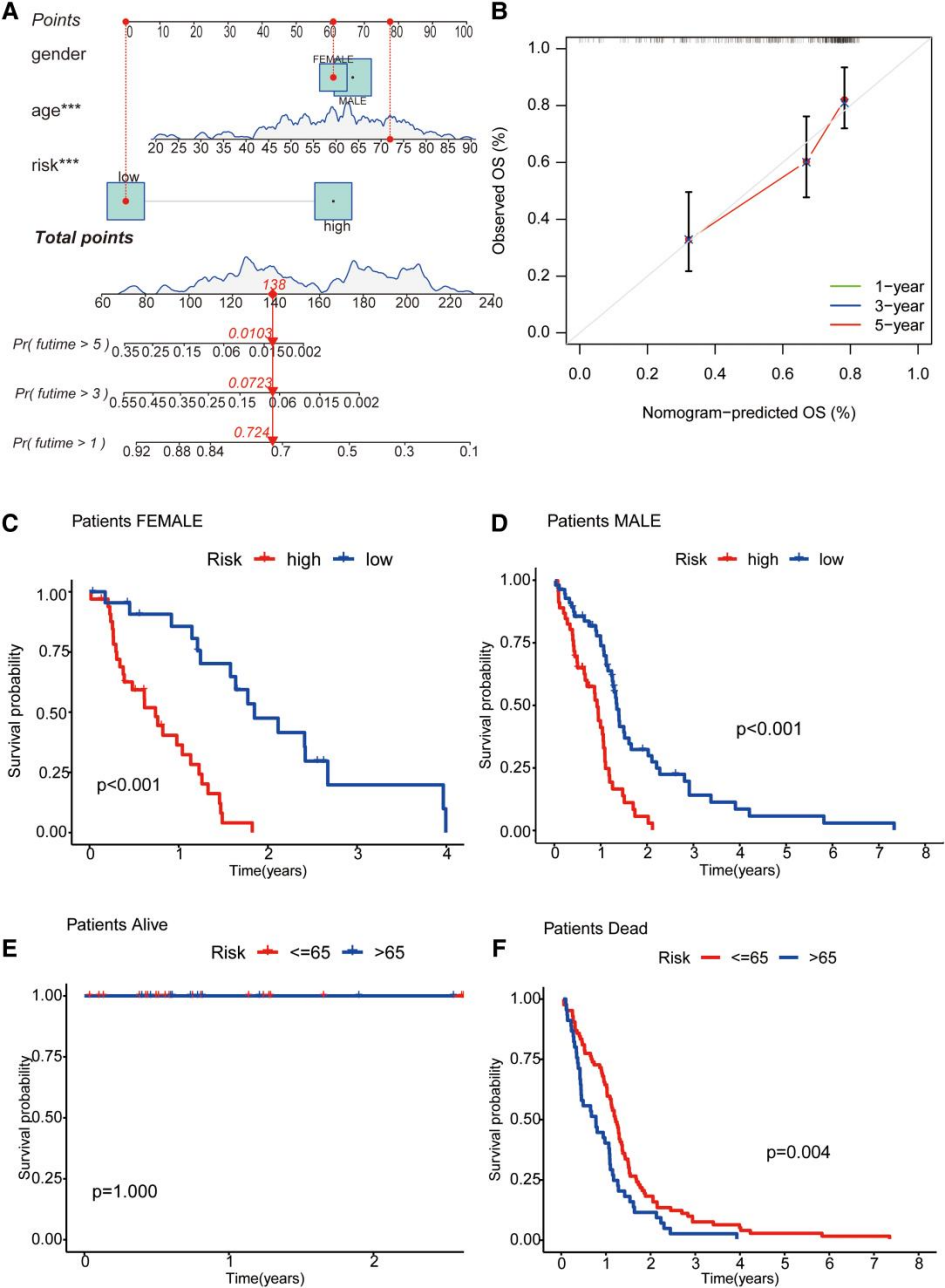
**Nomogram and clinical grouping verification of GTLncSig model.** (**A**) The risk score, age and gender are combined to construct a nomogram to predict the 1-year (0.724), 3-year (0.0723), and 5-year (0.0103) survival probability of GBM patients (*n* = 159), and it is getting less from 1 to 5 years. The nomogram calculates risk scores based on the Cox model’s coefficients. (**B**) The curve is used to estimate the accuracy of the nomogram. Calibration curves are generated for different time intervals (1, 3 and 5 years). (**C**) The consistency model is to predict the survival of female patients (*n* = 56, *P* < 0.001); (**D**) the survival of male patients (*n* = 103, *P* < 0.001); (**E**) the survival of patients alive (*n* = 30, *P* = 1); and (**F**) the survival of patients with dead (*n* = 129, *P* < 0.001). The log-rank test assesses whether there are significant differences in survival distributions between the groups, and the Kaplan–Meier estimates visualize the survival probabilities over time for each group.

For the sake of clinicopathological covariates, we chose a nomogram to build intuitive predictive models. Based on univariate and multivariate Cox regression analyses, our nomogram can be used to predict patients’ OS at 1, 3 and 5 years (Fig. 3A). At the same time, the predictions of 1-, 5- and 10-year OS from calibration plots are also good compared with our model (Fig. 3B). Next, we grouped GBM patients by gender and by survival state to compare the OS of the high-risk and low-risk groups and observed the model’s applicability in different groups. The results showed that in the gender group, the OS of high-risk GBM patients was significantly lower than that of low-risk patients in different clinical groups. In contrast, in the survival status group, the survival time of the alive group was longer than that of the dead group. That is, the model is suitable for patients with different clinical characteristics (*P* < 0.01; Fig. 3C–F).

### Discrimination assessment between high-risk and low-risk groups

We performed PCA to assess whether lncRNA could distinguish high-risk and low-risk patients accurately and efficiently. By comparing the PCA models of the mRNA, all genes, lncRNA and risk lncRNA, we found that among these four models, the highest model is the map of model risk lncRNA (Supplementary Fig. 5A).

### Differential expression analysis and functional annotation of GTLncSig-related genes

**Figure 4:**
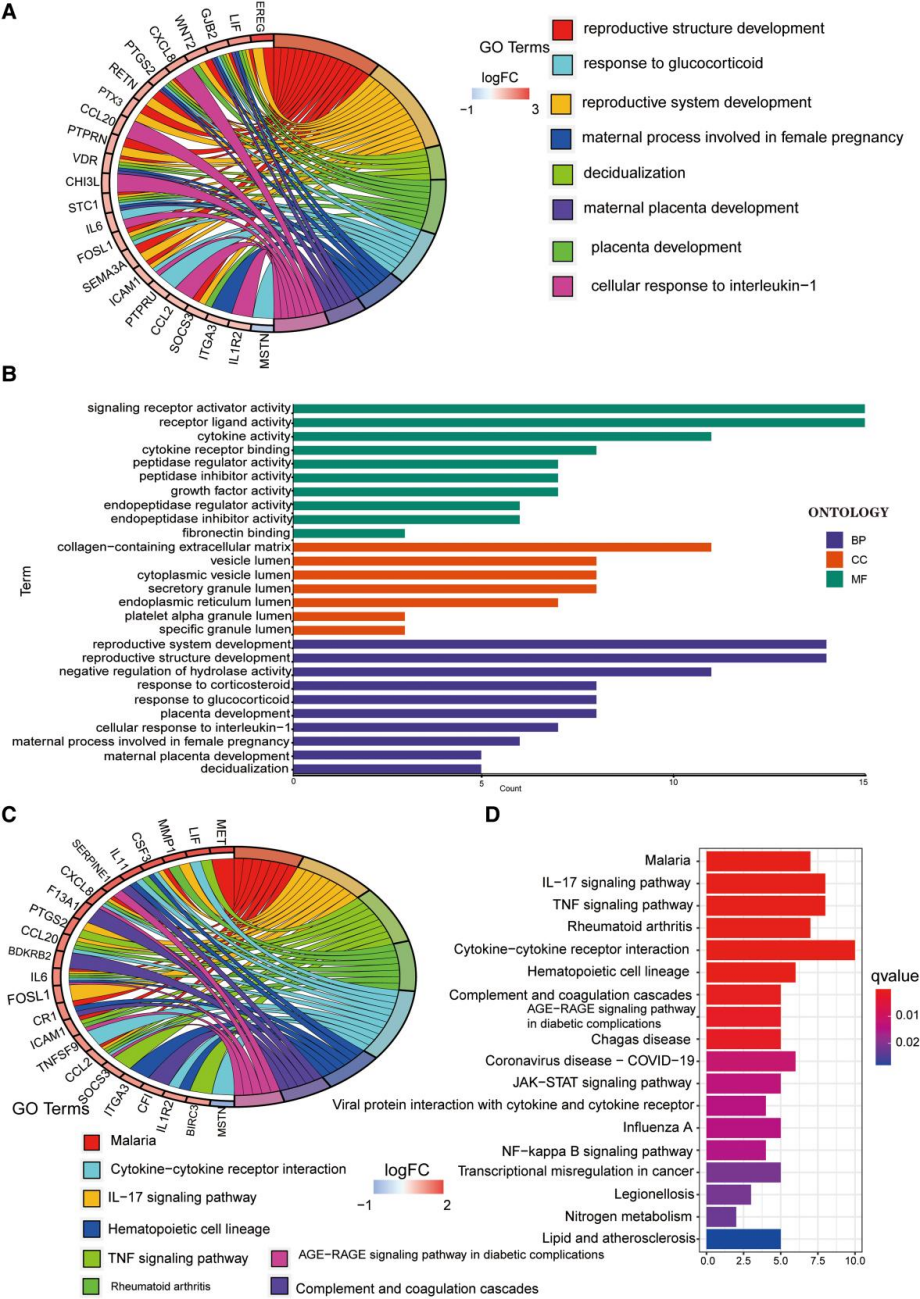
**GO/KEGG pathway for all differential genes between the high-risk and low-risk subgroups.** (**A** and **B**) The GO pathway analysis, including 23 genes. GO enrichment analysis is performed on a gene list, filters and visualizes enriched terms and generates circular visualizations to represent relationships between enriched terms and genes. (**C** and **D**) The result of the KEGG pathway.

We conducted a more comprehensive risk differential analysis to explore the specific BPs affected by risk. First, we screened out differentially expressed GTLncSig genes between the high-risk and low-risk groups. Then, we performed GO enrichment analysis on the above genes to observe which biological functions they are enriched in (Fig. 4A). The analysis revealed that these genes were mainly associated with signalling receptor activator activity and receptor–ligand activity in BPs. In addition, these genes are located primarily in the collagen-containing extracellular matrix in cellular components. The molecular function enrichment analysis data further indicated that most genes were associated with reproductive system development, reproductive structure and the development of negative regulation of hydrolase activity. We also visualized representative genes and GO terms (Fig. 4B). According to the results of KEGG, the related genes are mostly associated with the cytokine–cytokine receptor interaction pathway and show high correlations in the interleukin-17 signalling pathway, tumour necrosis factor (TNF) signalling pathway, malaria and rheumatoid arthritis (Fig. 4C). The connections between genes and the KEGG pathway are visualized in Fig. 4D.

### Exploring immune pathway enrichment of GTLncSig in GBM

The risk signals we constructed were all related to immune regulation, so we further analysed differences in immune function between patients in the high-risk and low-risk groups. Among them, according to the immune function heatmap in the training cohort, in high-risk, most immune phase functions were significantly upregulated, including Type_II_IFN_Reponse, Inflammation-promoting, T_cell_co-stimulation, HLA, Parainflammation and APC_co_stimulation and the other (Supplementary Fig. 5B). Almost all of them are highly expressed in the high-risk group and relatively low in the low-risk group, so they can be considered as high-risk factors. Then, whether it is in the testing or the entire cohort, it is consistent with the expression of the training cohort (Supplementary Fig. 5C and D).

### The TMB differential analysis of GTLncSig

**Figure 5:**
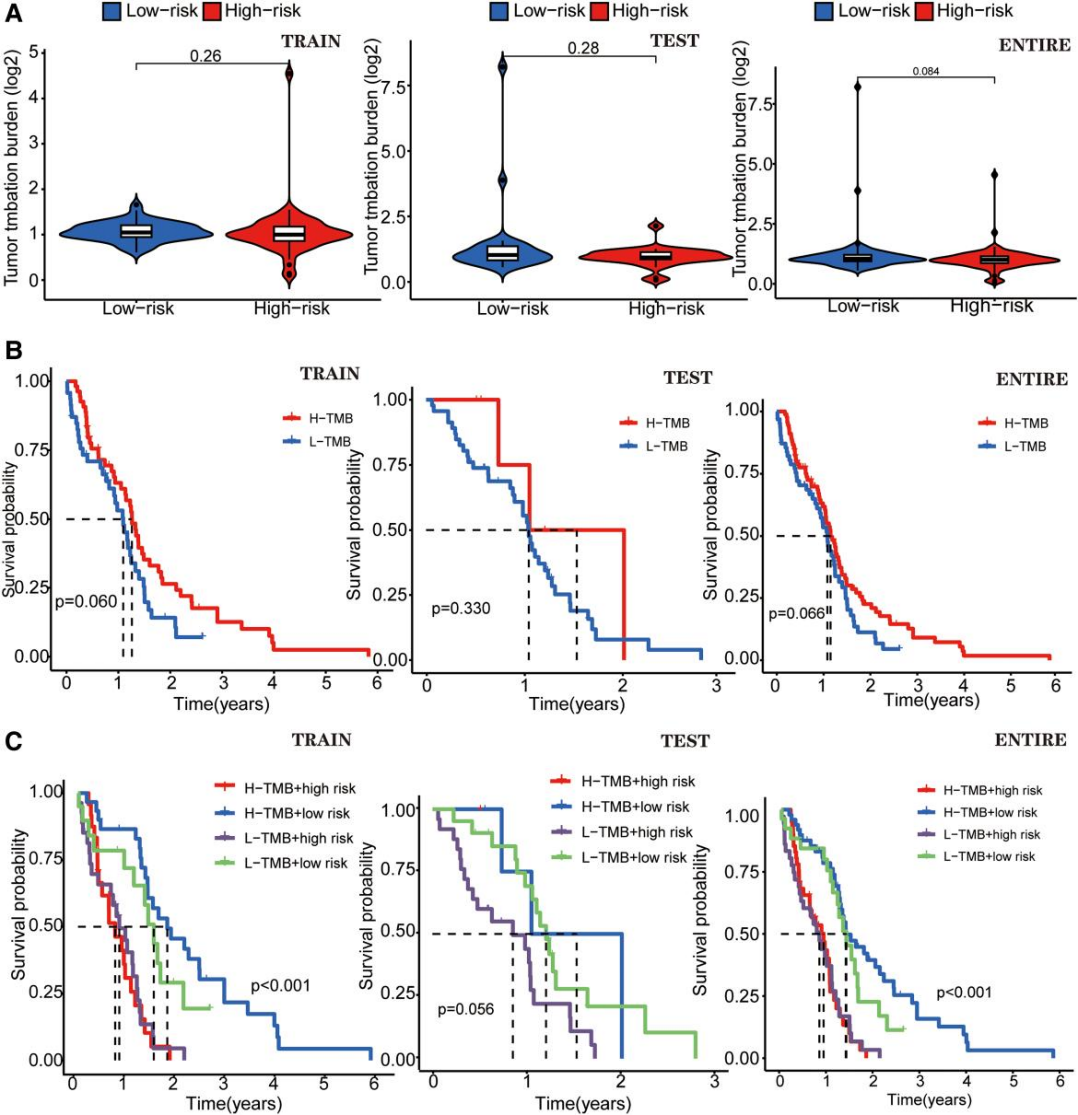
**A different analysis of TMB of GTLncRNAs.** (**A**) Boxplots and survival curves show no difference by using the *χ*^2^ test statistic, and the associated *P*-value (*P* > 0.05) in TMB analyses and visualizes the differences between the high-risk (*n* = 79) and low-risk (*n* = 80) subgroups. (**B**) Another survival curve illustrates the variability in survival time based on the TMB risk score. None of these curves exhibit statistical significance when assessed using the *χ*^2^ test statistic and the corresponding *P*-value (*P* > 0.05). (**C**) The survival of the TMB+ high-risk (*n* = 79) and low-risk (*n* = 80) groups in three different cohorts. In the training (*n* = 107) and the entire groups (*n* = 159), the results show differences (*P* < 0.001). The *χ*^2^ test statistic and the associated *P*-value are used to evaluate whether there are significant differences in survival experiences between different risk groups, and both groups illustrate that the H-TMB+ high-risk group has the most extended survival probability.

We compared the TMB in high-risk and low-risk groups of GTLncSig and found no difference in the training cohort (*P* = 0.26), the testing cohort (*P* = 0.28) and the entire cohort (*P* = 0.084; Fig. 5A). TMB survival curve results for the training cohort (*P* = 0.060), the test cohort (*P* = 0.330) and the entire cohort (*P* = 0.066; Fig. 5B) were statistically insignificant. We cannot say that the greater the tumour mutational burden, the higher the risk of glioma. We made a survival curve to visually analyse TMB, risk and survival. In both the entire cohort and training cohort (*P* < 0.001), the H-TMB+ low-risk subgroup had the longest survival, while the H-TMB+ high-risk subgroup had the shortest survival (Fig. 5C). However, based on previous results, the dose of H-TMB was insignificant. In the testing group, the L-TMB+ low-risk subgroup has the longest survival time, but this group has no significance (*P* > 0.05).

### Analysis of GTLncSig for immune escape and immunotherapy in GBM

**Figure 6:**
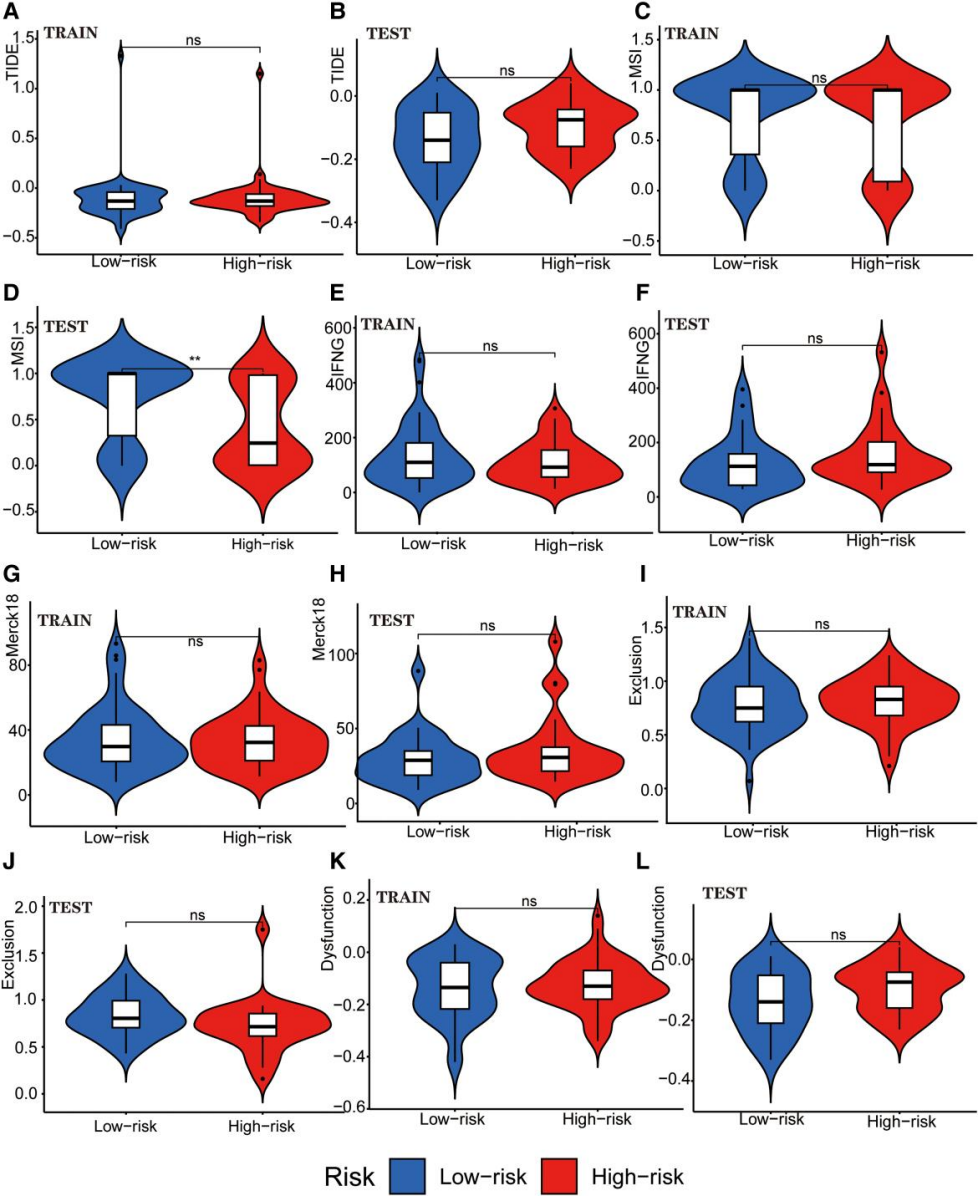
**Tumour immune escape and immunotherapy analysis for GTLncRNAs.** Analysis of the differences among TIDE (**A** and **B**), MSI (**C** and **D**), Interferon Gamma (IFNG, **E** and **F**), Merck 18 (**G** and **H**), Exclusion (**I** and **J**) and Dysfunction (**K** and **L**) in different risk subgroups. These methods are used to analyse and visualize the differences in TIDE scores between different risk groups for multiple TIDE score features (columns) using violin plots with added boxplots and statistical comparisons. Only one subgroup has significance: MSI in the testing group (*n* = 56, *P* < 0.01). The Wilcoxon rank-sum test (also known as the Mann–Whitney *U* test statistic) was used to compare whether the medians of two independent samples were significantly different. Significance labels (such as, ‘*’, ‘**’, ‘***’ and ‘ns’) are used to indicate the level of statistical significance, and there are no other specific statistical values. *P-value <0.05, **P-value <0.01, ***P value <0.001 and ns, no significance.

**Figure 7:**
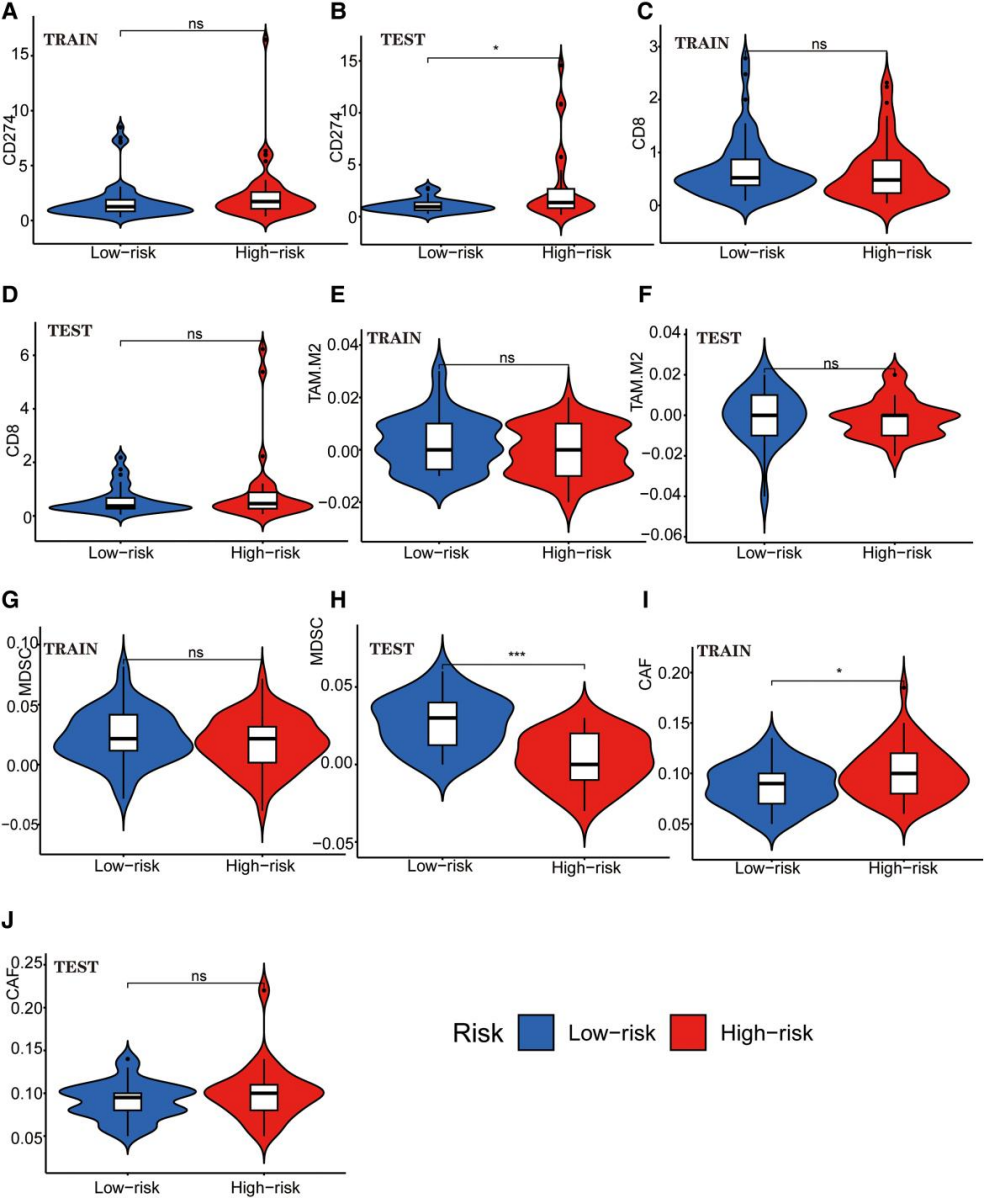
**Tumour immune escape and immunotherapy analysis for GTLncRNAs.** Analysis of the differences among CD274 (**A** and **B**), CD8 (**C** and **D**), TAM.M2 (**E** and **F**), MDSC (**G** and **H**) and CAF (**I** and **J**) in different risk subgroups. These methods are used to analyse and visualize the differences in TIDE scores between different risk groups for multiple TIDE score features (columns) using violin plots with added boxplots and statistical comparisons. Only a few subgroups have significance: CD274 in the testing group (*n* = 56, *P* < 0.05), MDSC in the testing group (*n* = 56, *P* < 0.001) and CAF in the training group (*n* = 103, *P* < 0.05). The Wilcoxon rank-sum test (also known as the Mann–Whitney *U* test statistic) was used to compare whether the medians of two independent samples were significantly different. Significance labels (such as, ‘*’, ‘**’, ‘***’ and ‘ns’) are used to indicate the level of statistical significance, and there are no other specific statistical values. *P–value <0.05, **P–value <0.01, ***P–value <0.001 and ns, no significance.

We examined the potential immune escape and response to immunotherapy of GTLncSig in the high-risk and low-risk subgroups. In addition, we analysed TIDE scores in the high-risk and low-risk groups to predict the therapeutic effect of immune checkpoint inhibition therapy. However, no significant difference was observed in TIDE scores between the high-risk and low-risk groups in the training or testing cohort (no significance, Fig. 6A and B). The lack of significance in the *P*-value precludes the prediction of patient response to immunotherapy. In MSI (Fig. 6C), there is no significance in the training group, while the testing group is significant (*P* < 0.01; Fig. 6D). We also analysed the importance of interferon gamma (IFNG) (Fig. 6E and F), Merck18 (Fig. 6G and H), Exclusion (Fig. 6I and J), dysfunction (Fig. 6K and L), CD274 (Fig. 7A and B), CD8 (Fig. 7C and D), TAM.M2 (Fig. 7E and F), MDSC (Fig. 7G) and CAF (Fig. 7I and J). Still, no significant differences were observed except for MDSC in the testing group (*P* < 0.001; Fig. 7H). Further research is needed to investigate the significance of MDSC.

### Screening of chemotherapeutic drugs for graphene therapy

**Figure 8:**
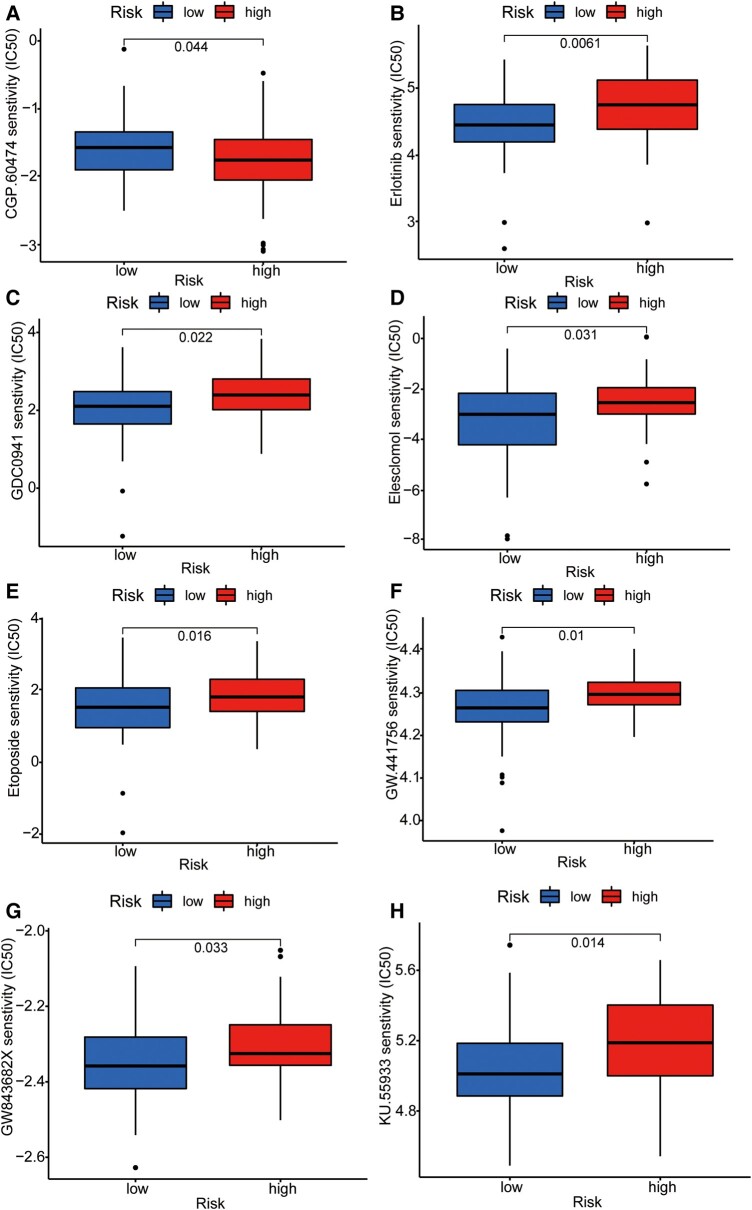
**Sensitivity analysis of anti-tumour drugs based on GTLncSig.** The analysis includes the differences in drug sensitivity (half-maximal inhibitory concentration values) between the risk groups for a list of predefined drugs. It involves pre-processing gene expression data, performing drug sensitivity predictions, conducting statistical tests and visualizing the results using boxplots with added statistical comparisons. Respectively, (**A**) CGP.60474, (**B**) Erlotinib, (**C**) GDC0941, (**D**) Elesclomol, (**E**) Etoposide, (**F**) GW.441756, (**G**) GW843682X, (**H**) KU.55933. The Wilcoxon rank-sum test statistic and the associated *P*-value are used to assess whether the distributions of sensitivity values for the high-risk (*n* = 79) and low-risk (*n* = 80) groups are significantly different. If the *P*-value is below 0.05, it suggests a statistically significant difference in drug sensitivities between the two risk groups.

**Figure 9:**
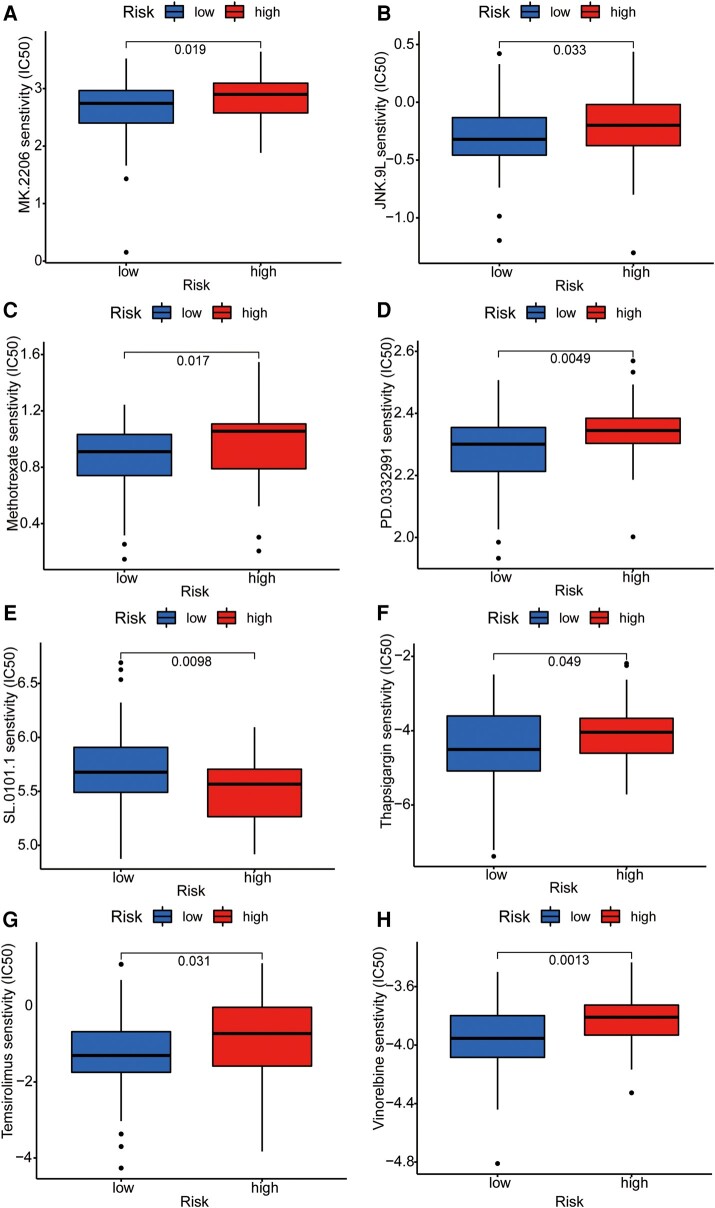
**Sensitivity analysis of anti-tumour drugs based on GTLncSig.** The analysis includes the differences in drug sensitivity (half-maximal inhibitory concentration values) between the risk groups for a list of predefined drugs. It involves pre-processing gene expression data, performing drug sensitivity predictions, conducting statistical tests and visualizing the results using boxplots with added statistical comparisons. Respectively, (**A**) MK.2206, (**B**) JNK.9L, (**C**) Methotrexate, (**D**) PD.0332991, (**E**) SL.0101.1, (**F**) Thapsigargin, (**G**) Temsirolimus, (**H**) Vinorelbine. The Wilcoxon rank-sum test statistic and the associated *P*-value are used to assess whether the distributions of sensitivity values for the high-risk (*n* = 79) and low-risk (*n* = 80) groups are significantly different. If the *P*-value is below 0.05, it suggests a statistically significant difference in drug sensitivities between the two risk groups.

To further integrate the signalling model with clinical graphene oxide treatment, we analysed the sensitivity of GTLncSig in all anticancer drugs and screened out the drugs with significant efficacy. We used the pRRophetic algorithm to estimate the sample’s response to treatment based on the half-maximal inhibitory concentration (IC50), which can be found in the Genomics of Drug Sensitivity in Cancer database. According to the algorithm, 31 compounds were screened, most of which had significantly different estimated half-inhibitory concentrations between the high-risk and low-risk subgroups. The high-risk subgroup was more sensitive to most of them. The figure below shows the 16 most common compounds (Figs 8 and 9) available for graphene oxide therapy in GBM patients (*P* < 0.05).

### Authenticate with the IMvigor210 immunotherapy model

We compared the gene analysis obtained from LASSO regression, and to verify the prognostic value of the GTLncSig model, we calculated the corresponding risk scores of patients in the IMvigor210 cohort by formula. We divided them into high-risk and low-risk groups. Unfortunately, there was no difference in survival between our high-risk and low-risk groups in IMvigor210 bladder cancer (*P* > 0.05; Supplementary Fig. 6A). And then, we validated it with ROC curves, which were still unpredictable (Supplementary Fig. 6B). The risk score of target genes was insignificant between different drug responses to immunotherapy for IMvigor210 bladder cancer (*P* = 0.24; Supplementary Fig. 6C).

### GBM stemness index analysis

The mRNAsi are a computational metric data based on gene expression. First, we compared the content of mRNAsi in normal tissues and GBM tumour tissues and found a significant difference (*P* = 0.002). The content in tumour tissues was much higher than that in normal tissues (Supplementary Fig. 6D). The survival probability of groups with different levels of mRNAsi was compared, but unfortunately we found no difference (*P* = 0.168; Supplementary Fig. 6E).

## Discussion

Bioinformatics has rapidly advanced in recent years, leading to the discovery of many reliable prognostic signatures based on lncRNAs to determine the prognosis of various malignancies.^37-40^ LncRNAs function in multiple ways, interacting with proteins, DNA and RNA and regulating different BPs,^41,42^ ultimately affecting the prognosis of cancer patients. For example, in GBM patients, the abnormal expression of certain specific lncRNAs in tumour cells can be used as diagnostic markers or potential drug targets.^43^

The field of tumour medicine has seen a recent advancement in the form of graphene oxide therapy, which offers a promising method for loading various therapeutic drugs, including anticancer drugs, insoluble drugs, antibiotics, antibodies, peptides, DNA, RNA and genes. Graphene offers ultra-high drug-loading efficiency due to its large surface area.^44^ Furthermore, with the ability of lncRNAs to serve as diagnostic markers and potential drug targets in GBM patients, the precision drug-targeted therapy utilizing graphene oxide as a carrier presents a novel direction for future research. GBM is the humans’ most common and aggressive malignant primary brain tumour.^45^ Our study highlights the significance of lncRNAs in assessing the prognosis of GBM patients and the potential role of tumour immune infiltration in the disease.

In this study, we first screened 298 lncRNAs associated with differentially expressed genes in GBM by analysing their Pearson correlation. Next, the univariate Cox analysis identified 221 GTLncRNAs related to OS and GBM prognosis. Next, by constructing a signal model using LASSO regression and multivariate Cox analysis, we identified four GTLncRNAs (AC011405.1, HOXC13-AS, LINC01127 and LINC0157) that were closely related to OS. To further validate this model, we plotted the ROC curve, *C*-index curve and nomogram and performed a PCA. Our results demonstrate that the risk score of this model can effectively and accurately predict and assess OS and function in GBM patients independently of other clinical signals. We found that age and risk score had a more significant correlation with OS, consistent across the training, testing and entire cohorts. These findings highlight the potential of using GTLncRNAs as prognostic markers for GBM and suggest that the signal model we developed could guide clinical decision-making. Future studies could focus on further validating our model and exploring the role of these GTLncRNAs in the progression and treatment of GBM.

In recent years, immunotherapy has become a popular focus of disease treatment due to the importance of immune system abnormalities in the development of many diseases.^46^ Despite the poor prognosis of GBM patients with standard treatments, including surgical resection, radiation therapy and chemotherapy, GBM is a malignant tumour closely associated with immunosuppression.^47^ To further explore the clinical value of our constructed signalling model, we screened differentially expressed genes in the high-risk and low-risk groups and performed GO enrichment analysis. The results revealed that three contents related to immune response, including BP level, cellular components and molecular functions, were closely related to genes. Therefore, we further analysed the differences in the immune function of GBM patients in the high-risk and low-risk groups and found significant differences in the activity of multiple immune functions between the two groups. In the high-risk groups, most immune-related functions were significantly upregulated, including APC_co_inhibition, Para-inflammation, Cytolytic_activity, Inflammation-promoting, T_cell_co-inhibition, Check-point, T_cell_co-stimulation, APC_co_stimulation and CCR. These immune-related functions may be important factors leading to suppressed immune function and poor prognosis in the high-risk subgroup of patients.

This study found that the high-risk GBM patients were less responsive to immune checkpoint inhibitors based on differences in TIDE scores and other immunotherapies. Therefore, they further evaluated potential drug treatments for patients with higher risk scores, selecting 16 compounds with different sensitivities from different risk groups. For example, CGP.60474 was more effective in the low-risk subgroup, whereas erlotinib was more effective in the high-risk subgroup. We also emphasized the importance of correctly using suitable compounds and leveraging the high drug-loading properties of graphene in a precise combination of gene therapy and drug therapy to improve treatment outcomes for GBM patients.^48^ AGAP2-AS1, LINC01503, LINC01127, HOXC13-AS and ZMIZ1-AS1 were expressed in IMvigor210 bladder cancer, but unexpectedly, high-risk scores were associated with high survival rates. In the Progenitor Cell Biology Consortium in the database, stemmed features represent the one-class logistic regression algorithm.^36^ In addition, we know that the value of mRNAsi is proportional to the degree of tumour differentiation.^49^ The difference between mRNAsi in normal tissue and GBM tumour tissue was also confirmed in the experiment. Still, the stem cell index was not significantly correlated with OS between the high-risk and low-risk subgroups, indicating that the degree of cancer progression was unrelated to OS.

Currently, the use of graphene oxide as a nanocarrier is still in the preliminary research stage; many questions remain, such as the effectiveness and safety of the function-modified graphene oxide nanocarriers and their distribution in tumour tissue. Graphene oxide is mainly used for loading small-molecule drugs but not biological macromolecules, such as DNA and proteins, and for conducting multiple studies on the potential harmful effects of graphene oxide outside the body, which are lacking in the body experimental data.^50^ In summary, there are still many problems in the study of graphene oxide, which requires the scientific research team to strengthen the integration of disciplines to solve difficulties.

Overall, our study identified four useful GTLncRNAs to construct the efficient and accurate signalling models independent of other clinical features to predict and assess the prognosis of GBM patients. In immune-related analyses, we found that these GTLncRNAs were highly expressed in the high-risk group and were relatively low in the low-risk group. Interestingly, TMB was not found to be associated with survival in GBM patients, and TIDE risk scores were not significantly different. Furthermore, the probability of bladder cancer expressed by the target gene in IMvigor210 did not show a significant difference in survival between the high-risk and low-risk subgroups. Finally, we conducted drug screening and identified 16 compounds that may be available for GBM treatment, with different sensitivities in different risk groups. The precise combination of gene therapy and drug therapy using these compounds, together with the ultra-high drug-loading properties of graphene, may hold the key to breaking through the complex treatment of GBM patients.

## Supplementary Material

fcad293_Supplementary_Data

## Data Availability

This published article and its supplementary materials include all data generated or analysed during this study.
