# Supplementary material for: Computational identification of long non-coding RNAs associated with graphene therapy in glioblastoma multiforme
Source: Brain Commun. 2023 Oct 25;6(1):fcad293. doi: 10.1093/braincomms/fcad293 (PMC10754320; doi:10.1093/braincomms/fcad293)
Supplement: fcad293_Supplementary_Data [file fcad293_supplementary_data.pdf]

Supplementary material

Computational identification of lncRNAs associated with graphene therapy in glioblastoma multiforme

Supplementary Fig. 1……………………………………………………………………………… 2

Supplementary Fig. 2……………………………………………………………………………… 3

Supplementary Fig. 3……………………………………………………………………………… 4

Supplementary Fig. 4……………………………………………………………………………… 7

Supplementary Fig. 5……………………………………………………………………………… 11

Supplementary Fig. 6……………………………………………………………………………… 13

Supplementary Table 1…………………………………………………………………………… 16

Supplementary Table 2…………………………………………………………………………….. 19

Supplementary Table 3…………………………………………………………………………….. 24

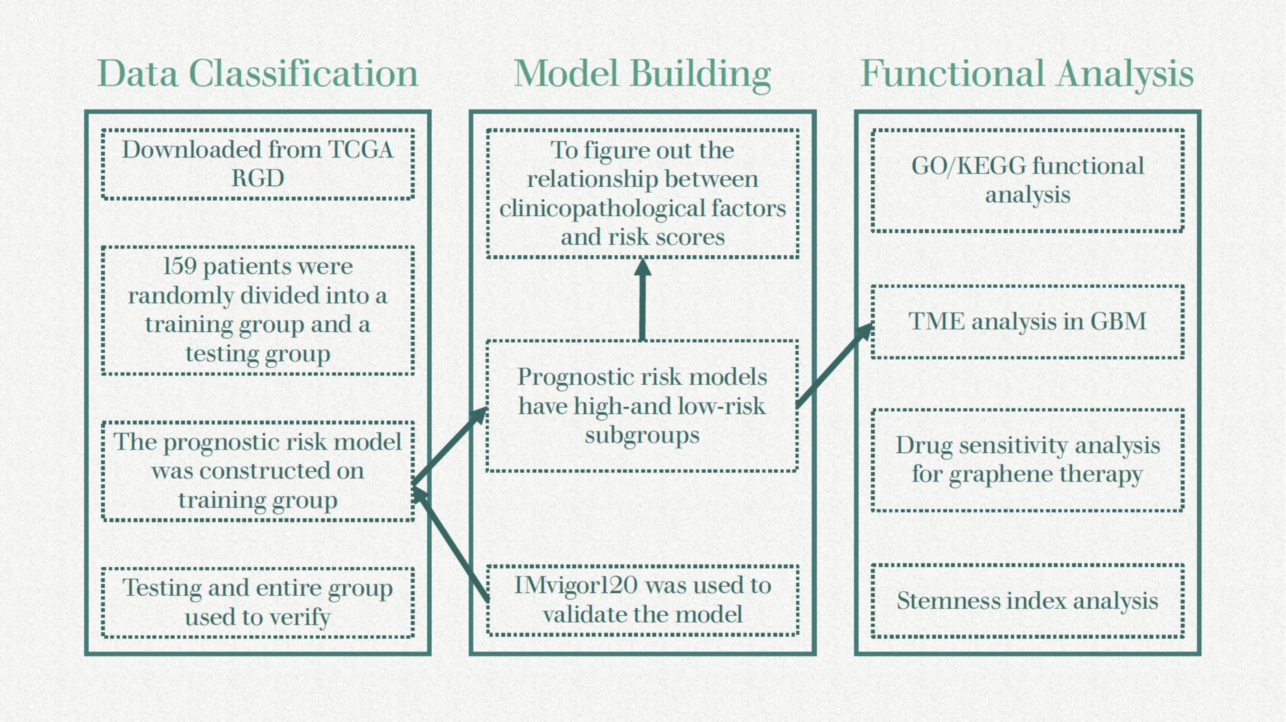

Supplementary Fig. 1. The flowchart of this study.

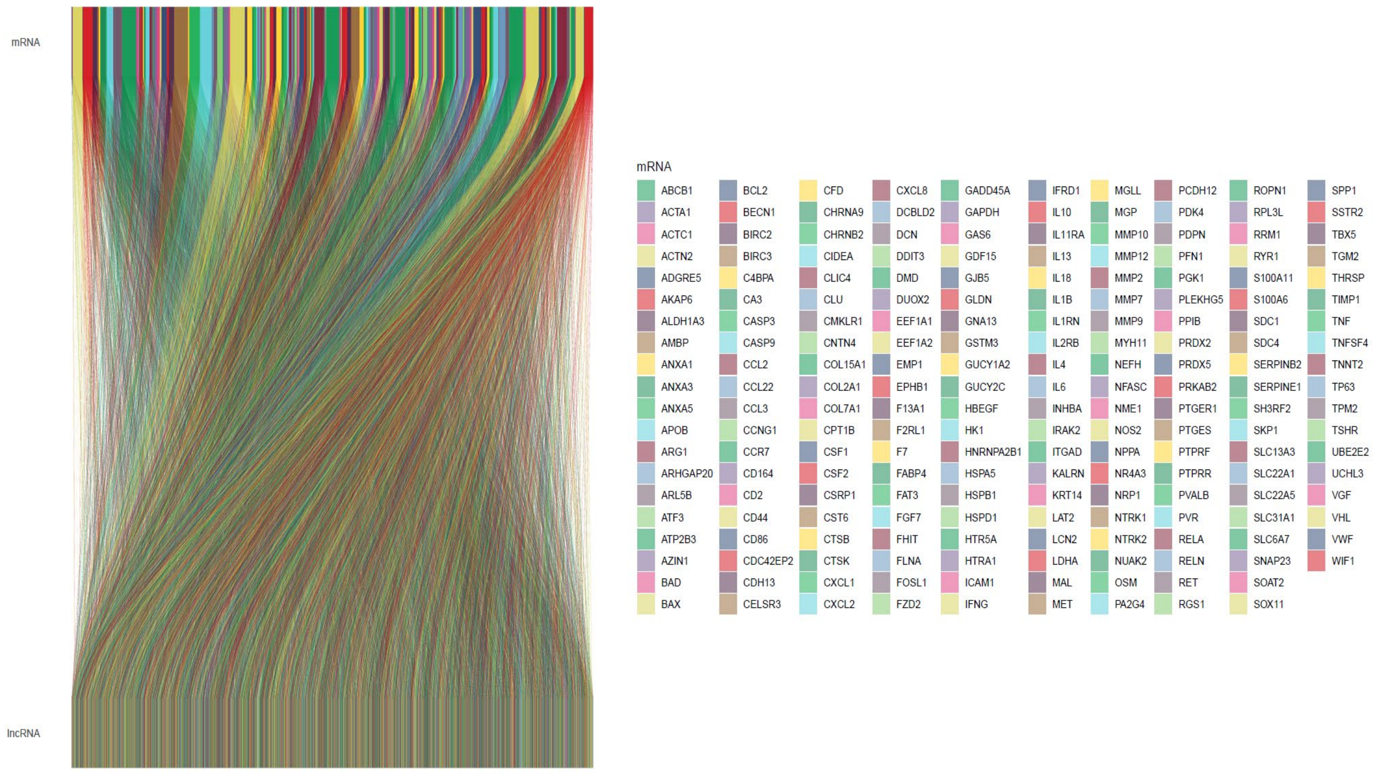

Supplementary Fig. 2. Visualization of target genes and Graphene Therapy-related LncRNAs. Sankey diagram showed the expression correlation between genes and Graphene Therapy-related LncRNAs. The Pearson correlation coefficient and its associated p-value are used to determine the strength and direction of the correlation between the expression of a given Graphene Therapy-related LncRNAs and mRNAs pair, and the calculated correlation coefficient meets the correlation filter and the p-value is below 0.5, the correlation is considered statistically significant.

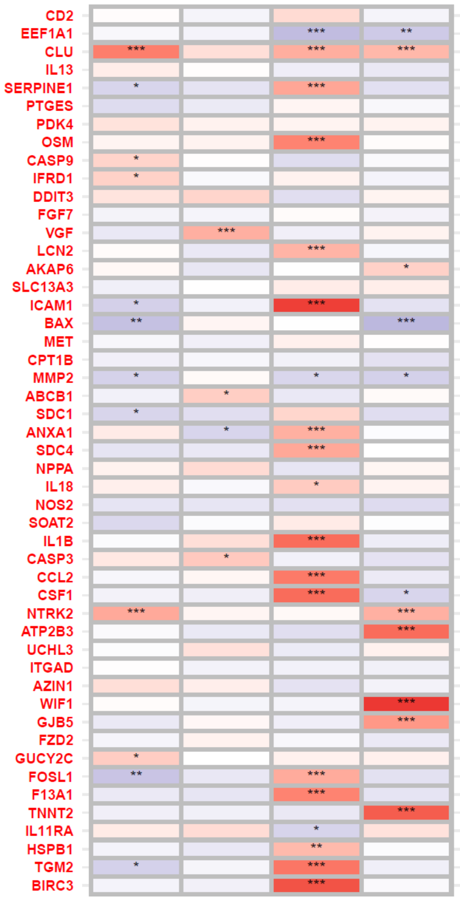
CD2
EEF1A1
CLU
IL13
SERPINE1
PTGES
PDK4
OSM
CASP9
IFRD1
DDIT3
FGF7
VGF
LCN2
AKAP6
SLC13A3
ICAM1
BAX
MET
CPT1B
MMP2
ABCB1
SDC1
ANXA1
SDC4
NPPA
IL18
NOS2
SOAT2
IL1B
CASP3
CCL2
CSF1
NTRK2
ATP2B3
UCHL3
ITGAD
AZIN1
WIF1
GJB5
FZD2
GUCY2C
FOSL1
F13A1
TNNT2
IL11RA
HSPB1
TGM2
BIRC3

Continued

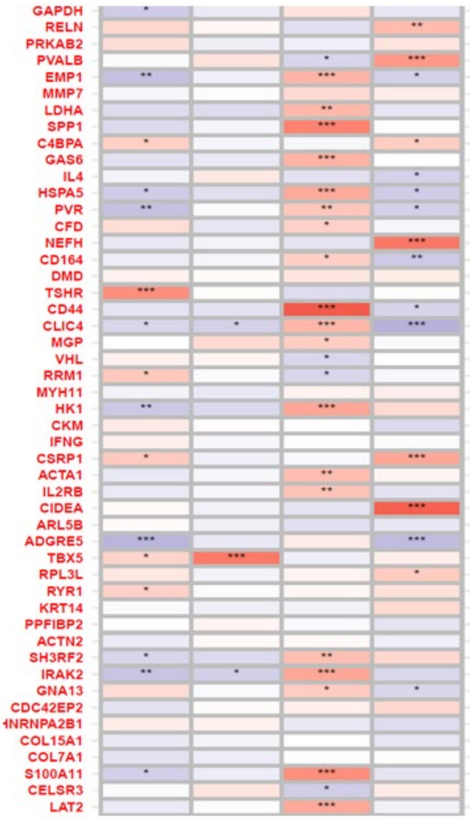

Continued

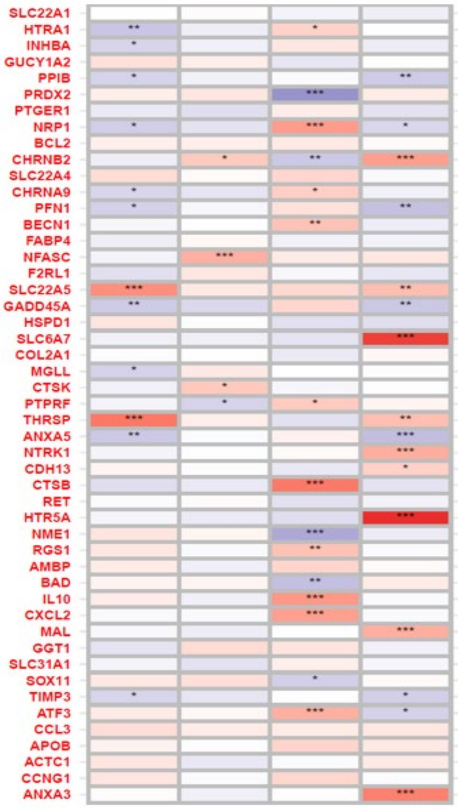

Continued

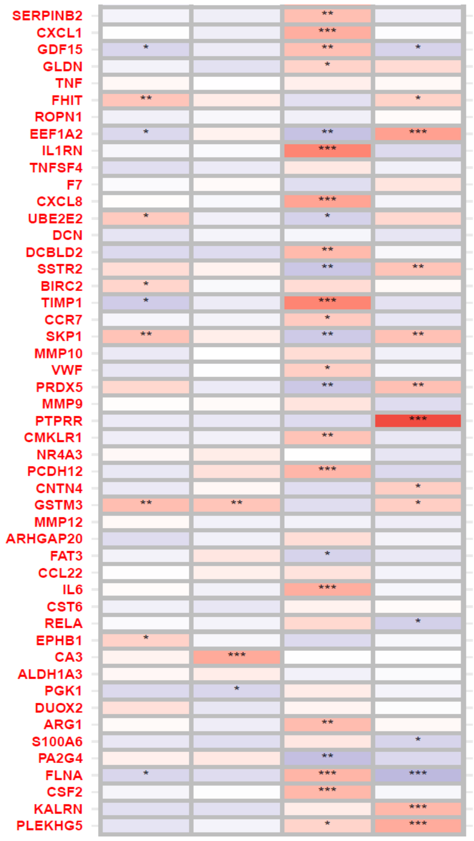

Continued

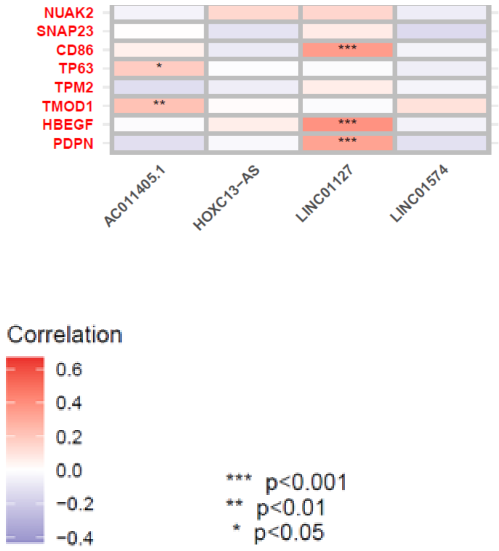

Supplementary Fig. 3. The prognostic risk model was established by lasso regression analysis, and the heat map illustrates the expression correlation between Graphene Therapy-related LncRNAs and genes.

Correlation analysis between lncRNA expressions and mRNA expressions. For each combination of

lncRNA and mRNA, the Pearson correlation coefficient is calculated. 4 Graphene Therapy-related LncRNAs showed a connection with genes in glioblastoma multiforme patients (n=159), especially the LINC01127 showed a highly related relationship. The correlation coefficient (cor) and p-value (pvalue) are extracted from the correlation test.

A

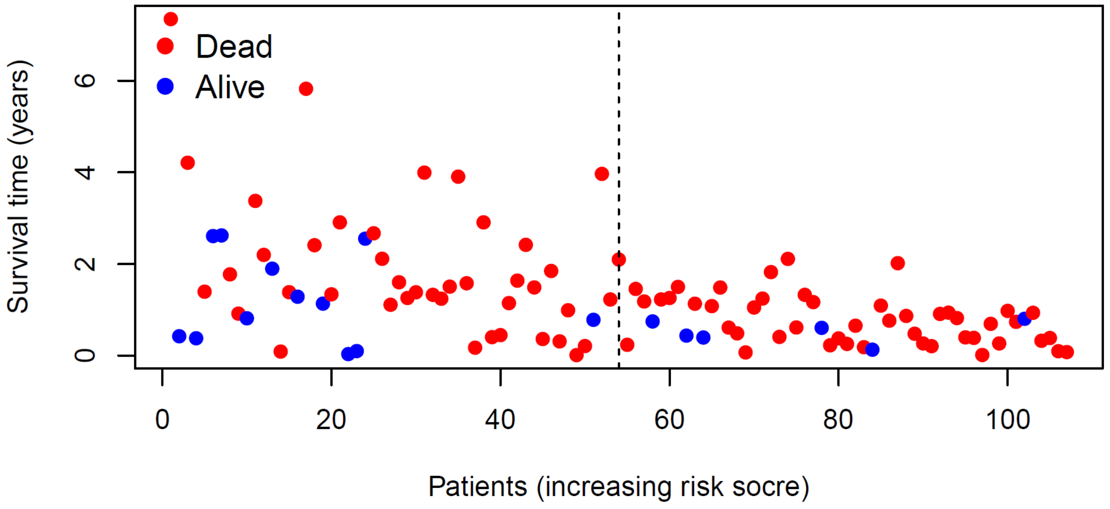

B

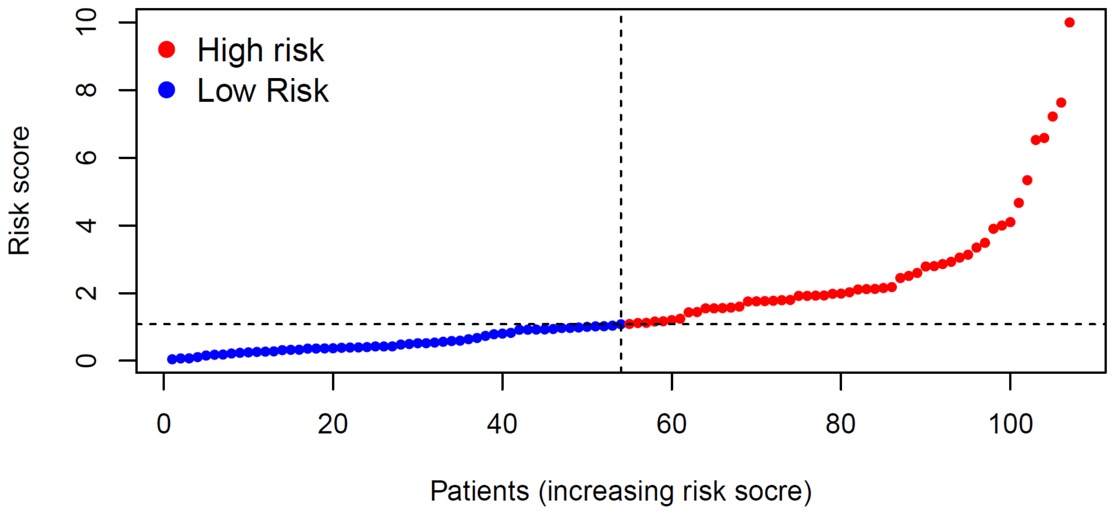

C

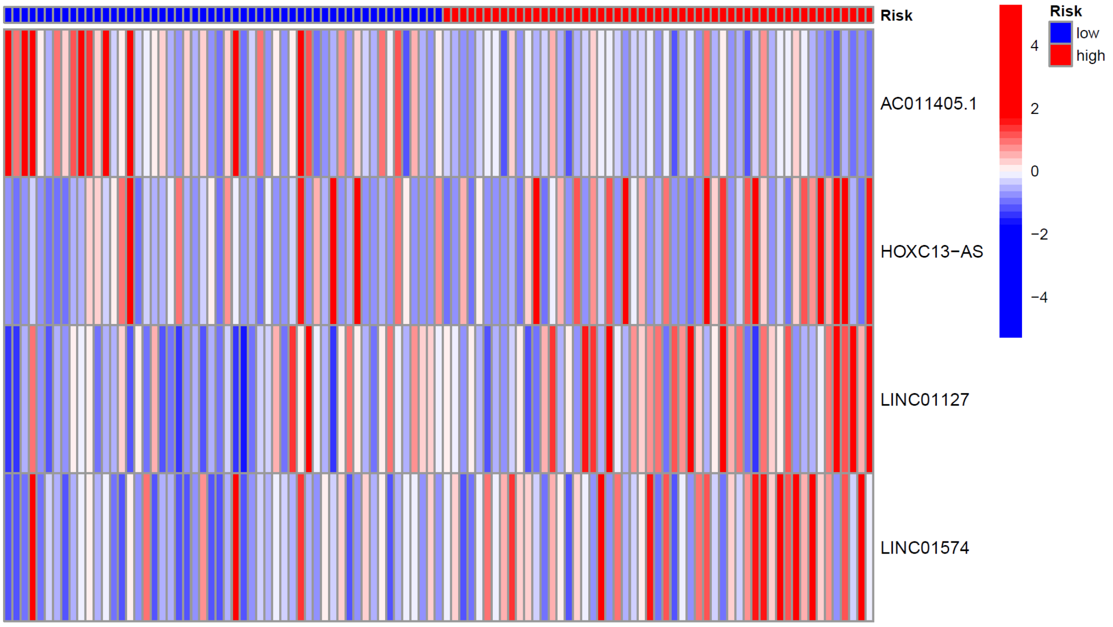
Risk
AC011405.1
HOXC13−AS
LINC01127
LINC01574
4
2
0
−2
−4
Risk
low
high

D

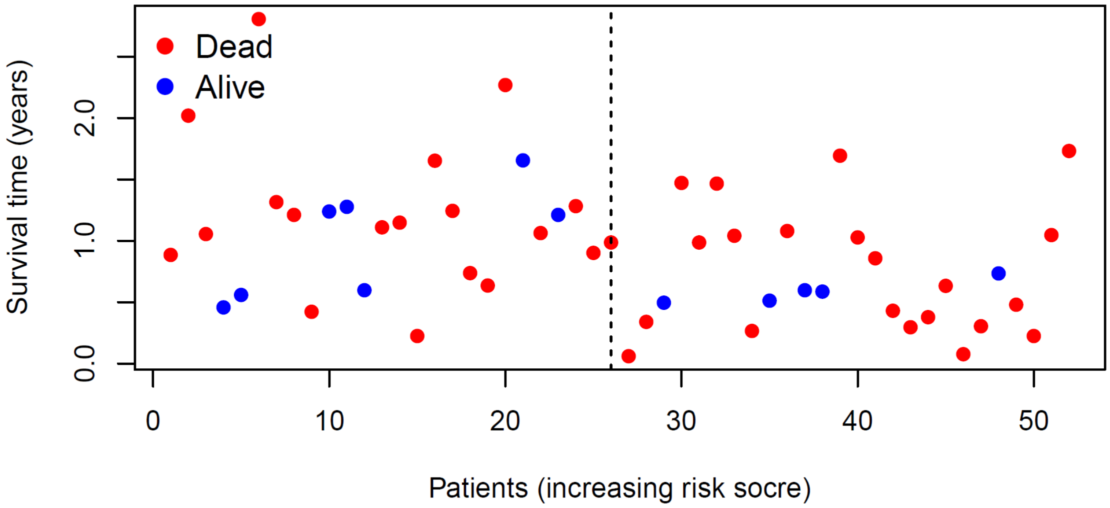
Dead
Alive
Survival time (years)
0.0
1.0
2.0
0
10
20
30
40
50
Patients (increasing risk socre)

E

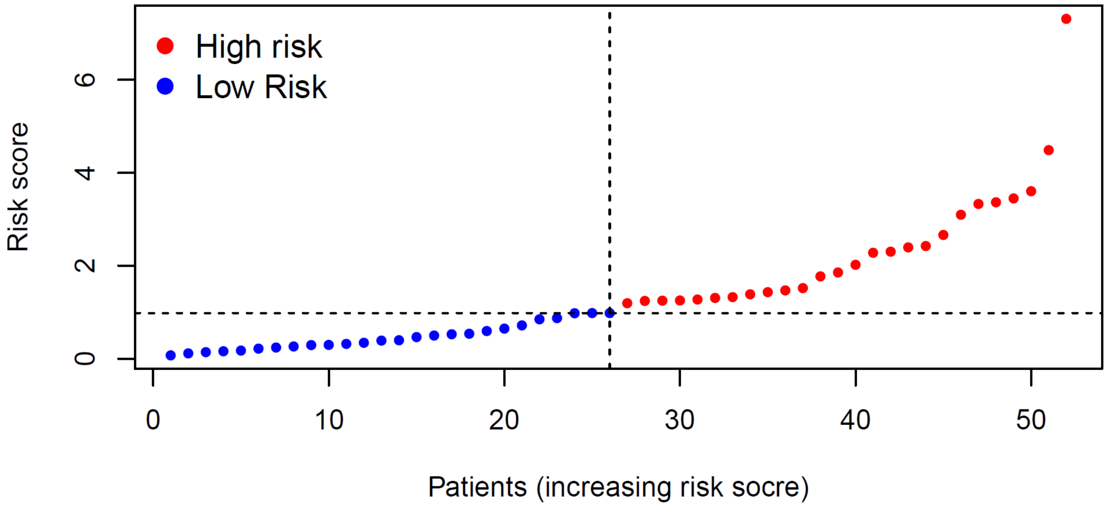
High risk
Low Risk
Risk score
0
2
4
6
0
10
20
30
40
50
Patients (increasing risk socre)

F

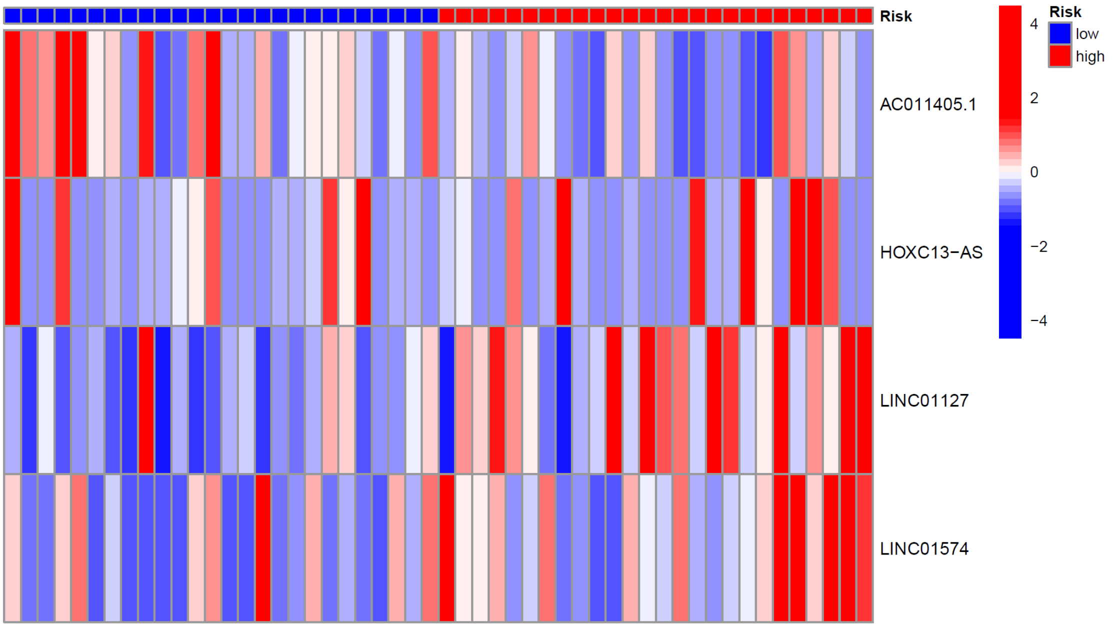
Risk
AC011405.1
HOXC13−AS
LINC01127
LINC01574
4
2
0
−2
−4
Risk
low
high

G

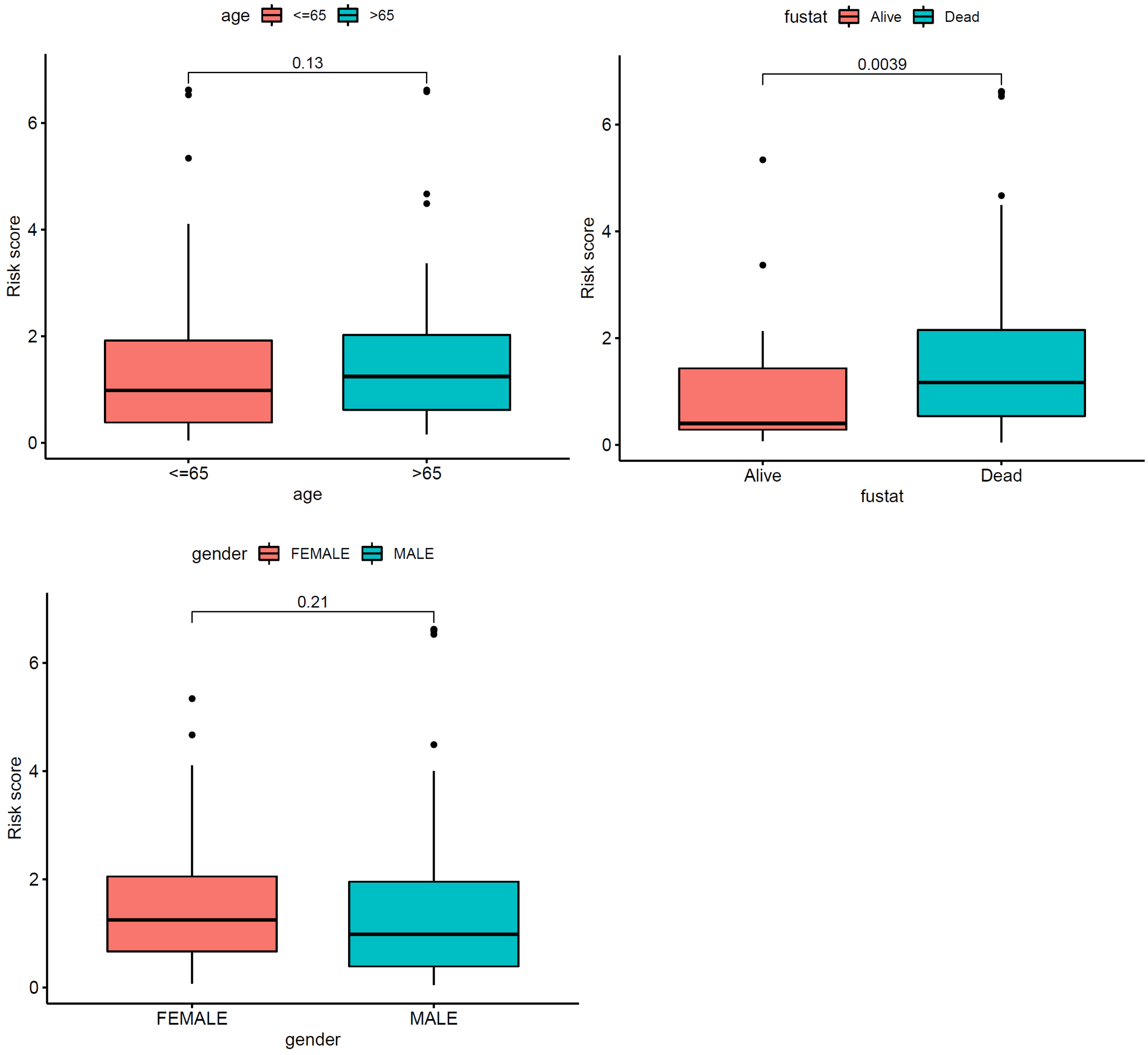
age
<=65
>65
0.13
Risk score
6
4
2
0
<=65
>65
age
fustat
Alive
Dead
0.0039
Risk score
6
4
2
0
Alive
Dead
fustat
gender
FEMALE
MALE
0.21
Risk score
6
4
2
0
FEMALE
MALE
gender

Supplementary Fig. 4. Construction of Graphene Therapy-related LncRNA-based gene Signatures risk model in different cohorts and the relationship between clinicopathological factors and risk scores

(A, B) The risk score plot visualizes the distribution of risk scores and survival status in the training cohort (n=107). Patients in the training cohort were divided into risk groups on the median of the risk scores by Kaplan-Meier survival curve analysis. The line drawn at the point where the high-risk samples begin helps to restrict the two groups. The other presents the survival status of samples over time. By associating colors with the survival status (red for deceased, blue for alive), the plot helps to illustrate the survival outcomes based on risk scores. (C) The heatmap visualization adds depth to understanding risk score distribution and relationships and shows the expression of Graphene Therapy-related LncRNAs in the training cohort (n=107). HOXC13−AS, LINC01127, and LINC01574 are all highly expressed in the high-risk group (n=53), In the low-risk group (n=54), the expression is lower. (D, E) Survival status and risk scores in the testing cohort (n=52). (F) In the heatmap of the testing cohort (n=52), the result remains the same. (G) Correlation analysis investigates the relationship between the risk score and various clinical variables for patients (n=159). There are no significant relationship between the risk score and age (p=0.13) and gender (p=0.21), only a correlation with survival state (p=0.0039).

A

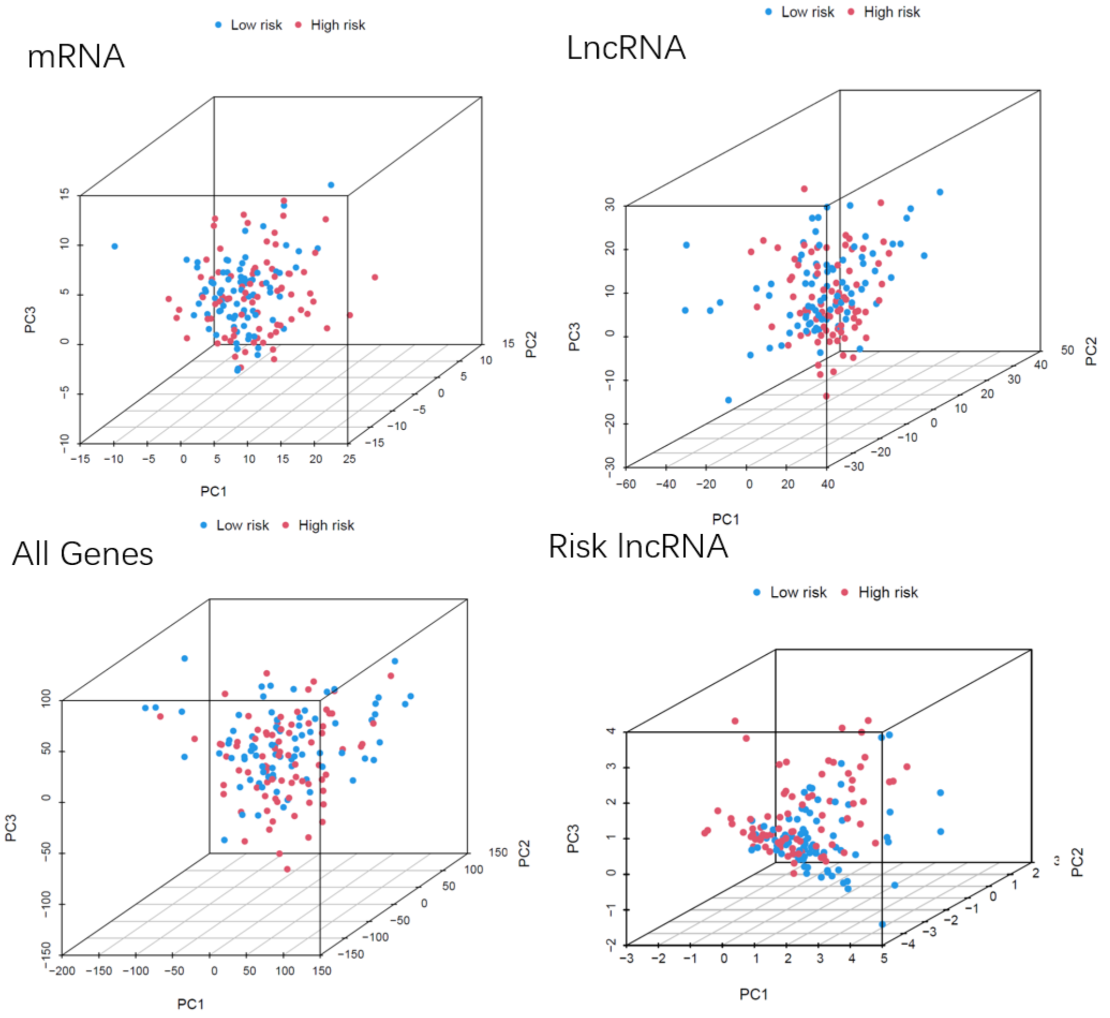
mRNA
Low risk
High risk
PC1
PC2
PC3
LncRNA
Low risk
High risk
PC1
PC2
PC3
All Genes
Low risk
High risk
PC1
PC2
PC3
Risk lncRNA
Low risk
High risk
PC1
PC2
PC3

B

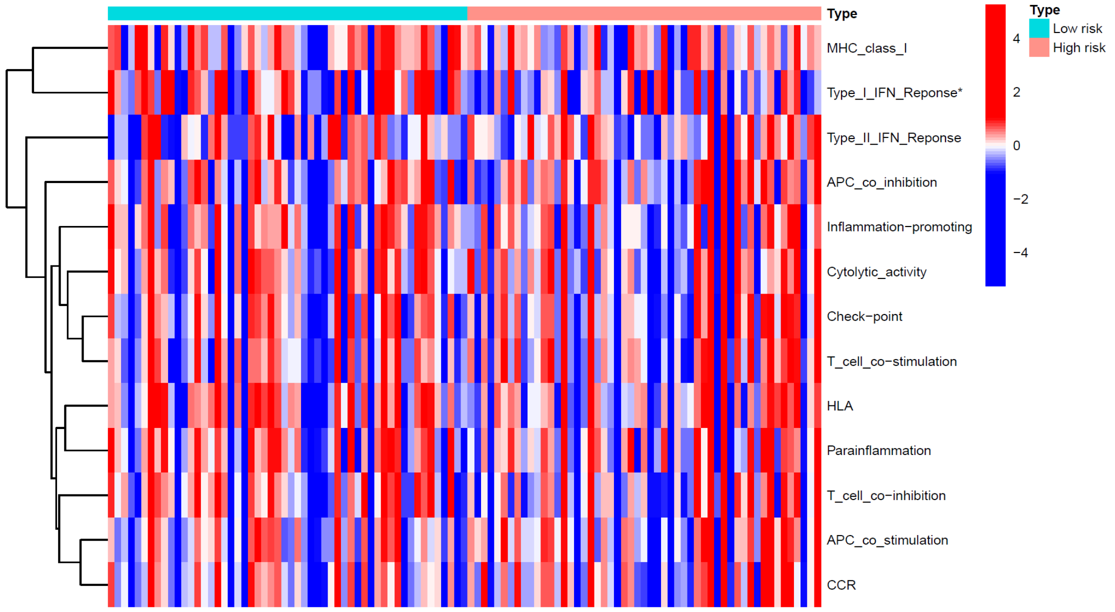
Type
MHC_class_I
Type_I_IFN_Reponse*
Type_II_IFN_Reponse
APC_co_inhibition
Inflammation−promoting
Cytolytic_activity
Check−point
T_cell_co−stimulation
HLA
Parainflammation
T_cell_co−inhibition
APC_co_stimulation
CCR
4
2
0
−2
−4
Type
Low risk
High risk

C

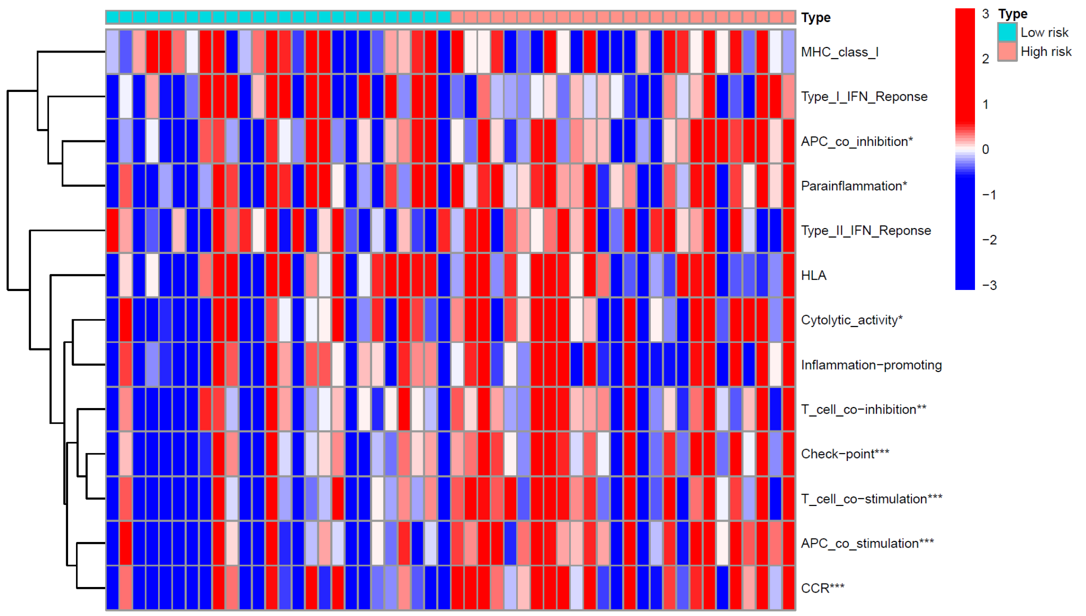

D

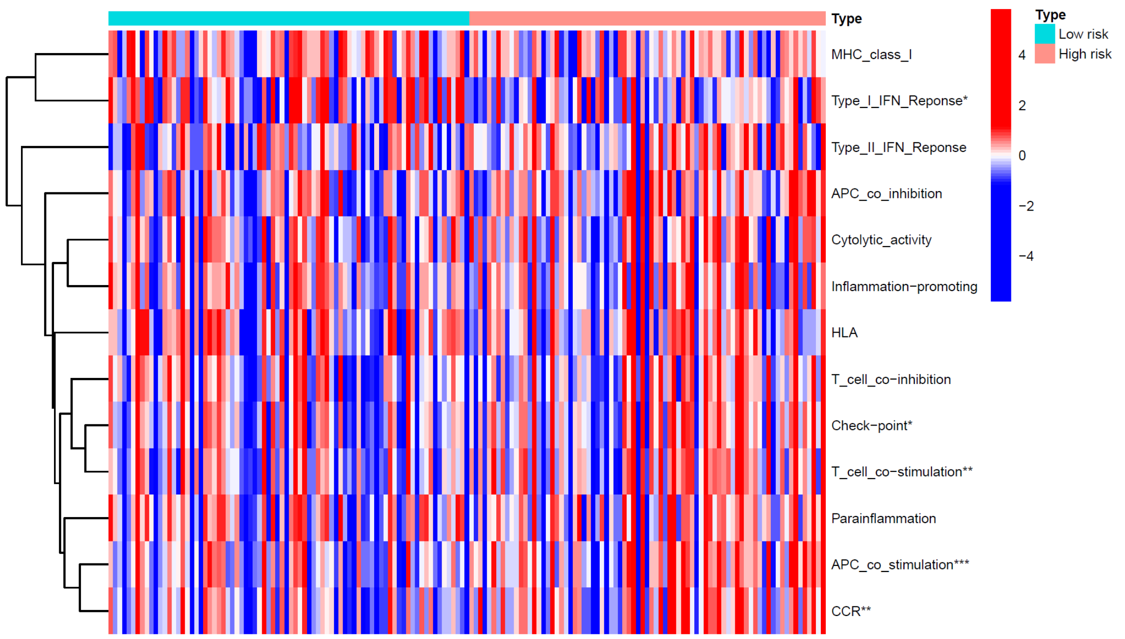

Supplementary Fig. 5. Three-dimensional principal component analysis (3d-PCA) and heatmap. (A) 3d-PCA is used to evaluate and compare the discrimination of mRNAs, all genes, lncRNAs, and risk lncRNAs in two subgroups. Perform PCA analysis on gene expression data and visualize the results in a 3D scatter plot. The factor with better discrimination would be a more valuable factor. (B) Heatmap performed by Single-sample Gene Set Enrichment Analysis (ssGSEA) analysis normalizes scores, identifies differentially expressed genes using Wilcoxon tests, and provides insights into the

differential expression patterns of genes between low and high-risk groups based on ssGSEA scores in the training cohort (n=107) in the high-risk group (n=53), most immune phase functions were significantly upregulated, including Type_II_IFN_Reponse, Inflammation-promoting, T_cell_co-stimulation, HLA, Parinflammation, and APC_co_stimulation, etc. (C, D) Heatmap of immune function in the testing (n=52) and entire cohort (n=159).

A

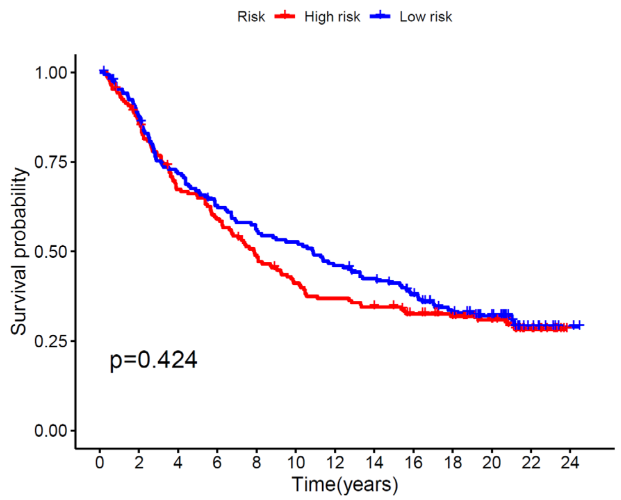

B

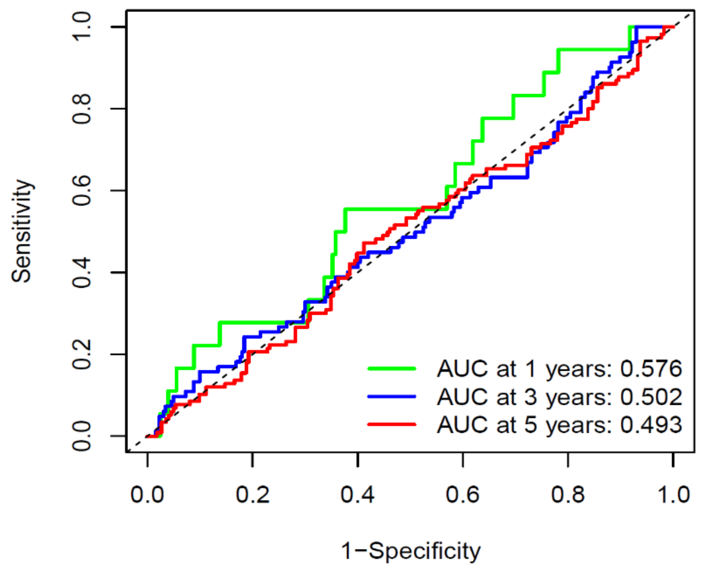

C

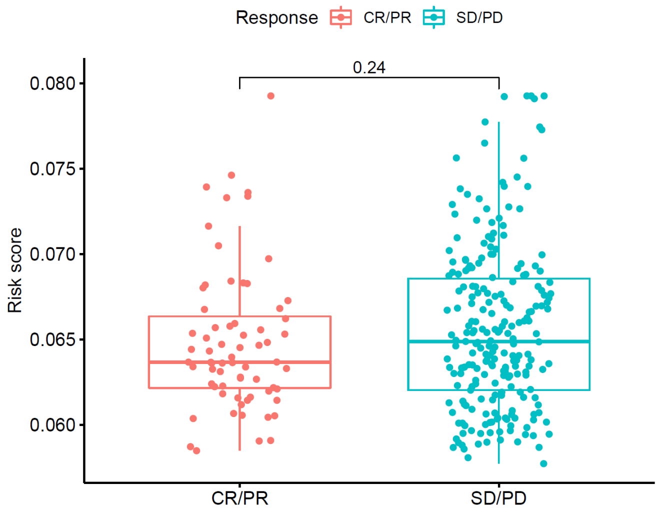
Response CR/PR SD/PD
0.24
Risk score
0.080
0.075
0.070
0.065
0.060
CR/PR
SD/PD

D

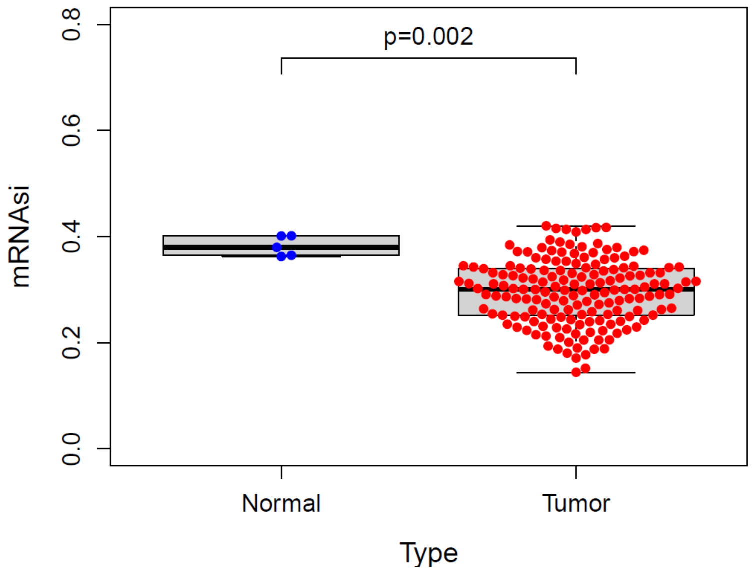
p=0.002
mRNAsi
0.0
0.2
0.4
0.6
0.8
Normal
Tumor
Type

E

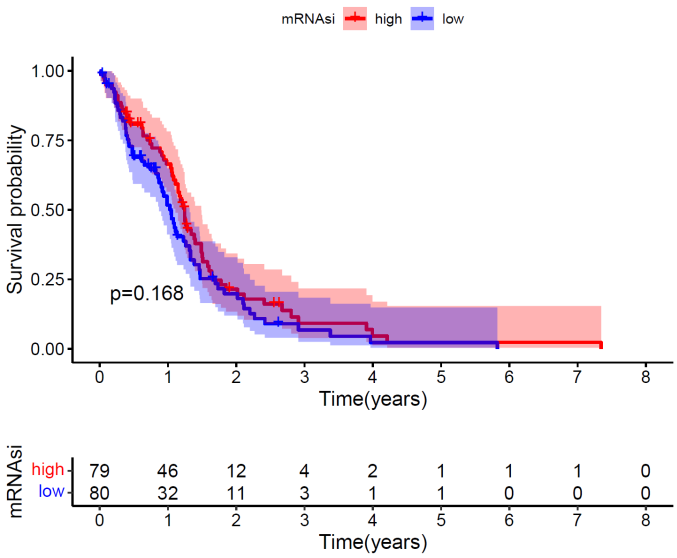
mRNAsi high low
Survival probability
1.00
0.75
0.50
0.25
0.00
p=0.168
0 1 2 3 4 5 6 7 8
Time(years)
mRNAsi
high 79 46 12 4 2 1 1 1 0
low 80 32 11 3 1 1 0 0 0
0 1 2 3 4 5 6 7 8
Time(years)

Supplementary Fig. 6. Validation with the IMvigor210 immunotherapy model and stemness index analysis.

(A) In the IMvigor210 model, higher-risk scores have higher survival rates, but p=0.424 has no significance in the high (n=79) and low-risk (n=80) groups. (B) The Receiver Operating Characteristic curve, which helps assess the predictive performance of a risk score in classifying high and low-risk groups in terms of survival outcomes, is poor. (C) There are no significant differences between responses to different immunotherapy drugs and no statistical significance (p=0.24). P-values are used for pairwise comparisons between different levels of the clinical variable. (D) A Wilcoxon rank-sum test compares mRNAsi between tumor and standard samples in a cancer dataset and the p-value of the Wilcoxon rank-sum test is displayed in the plot (p=0.002). (E) Wilcoxon rank-sum test is performed to explore the potential association between the "mRNAsi" variable and various clinical variables, and there are no differences in overall survival between high (n=79) and low (n=80) mRNAsi groups (p<0.05).

ID	Gender	ID	Gender	ID	Gender	ID	Gender	ID	Gender
TCGA-12-1091	FEMALE	TCGA-14-0740	MALE	TCGA-12-1597	FEMALE	TCGA-06-0189	MALE	TCGA-06-0164	MALE
TCGA-02-0266	MALE	TCGA-28-1757	MALE	TCGA-08-0244	MALE	TCGA-RR-A6KC	MALE	TCGA-12-3644	FEMALE
TCGA-08-0516	MALE	TCGA-19-0955	MALE	TCGA-32-1977	FEMALE	TCGA-14-0866	MALE	TCGA-28-1760	MALE
TCGA-08-0521	MALE	TCGA-06-0402	MALE	TCGA-06-5859	MALE	TCGA-12-0707	MALE	TCGA-26-1440	MALE
TCGA-08-0524	FEMALE	TCGA-08-0345	FEMALE	TCGA-28-2506	FEMALE	TCGA-12-5299	FEMALE	TCGA-32-2616	FEMALE
TCGA-02-0011	FEMALE	TCGA-08-0385	MALE	TCGA-41-4097	FEMALE	TCGA-19-1790	MALE	TCGA-12-0654	FEMALE
TCGA-02-0010	FEMALE	TCGA-76-6283	FEMALE	TCGA-06-0157	FEMALE	TCGA-27-2524	MALE	TCGA-16-1055	MALE
TCGA-06-A5U0	FEMALE	TCGA-28-5207	MALE	TCGA-28-5218	MALE	TCGA-06-0412	FEMALE	TCGA-02-0038	FEMALE
TCGA-06-2570	FEMALE	TCGA-06-0644	MALE	TCGA-06-0195	MALE	TCGA-06-0160	FEMALE	TCGA-41-3915	MALE
TCGA-32-1973	MALE	TCGA-06-0188	MALE	TCGA-12-0691	MALE	TCGA-06-1801	FEMALE	TCGA-19-1787	MALE
TCGA-15-1444	MALE	TCGA-28-5213	MALE	TCGA-08-0348	MALE	TCGA-12-1094	MALE	TCGA-27-2519	MALE
TCGA-06-5416	FEMALE	TCGA-06-2563	FEMALE	TCGA-32-2491	MALE	TCGA-32-4211	MALE	TCGA-19-0957	FEMALE
TCGA-14-1456	MALE	TCGA-06-0876	FEMALE	TCGA-14-1459	FEMALE	TCGA-19-5958	MALE	TCGA-02-0337	MALE
TCGA-06-2566	FEMALE	TCGA-32-1980	MALE	TCGA-08-0509	MALE	TCGA-19-5960	MALE	TCGA-08-0512	MALE
TCGA-06-2569	FEMALE	TCGA-06-5410	FEMALE	TCGA-26-1439	MALE	TCGA-02-0107	MALE	TCGA-12-0656	FEMALE
TCGA-12-0773	MALE	TCGA-19-1392	FEMALE	TCGA-27-2523	MALE	TCGA-02-0006	FEMALE	TCGA-06-6389	FEMALE
TCGA-32-4208	MALE	TCGA-06-0173	FEMALE	TCGA-76-6282	MALE	TCGA-08-0529	FEMALE	TCGA-76-6664	FEMALE
TCGA-02-0014	MALE	TCGA-RR-A6KA	FEMALE	TCGA-02-0075	MALE	TCGA-02-0111	MALE	TCGA-14-0781	MALE
TCGA-02-0271	MALE	TCGA-06-0210	FEMALE	TCGA-19-1390	FEMALE	TCGA-27-1834	MALE	TCGA-02-0052	MALE
TCGA-02-0087	FEMALE	TCGA-26-5135	FEMALE	TCGA-06-0137	FEMALE	TCGA-14-1829	MALE	TCGA-12-0618	MALE
TCGA-02-0026	MALE	TCGA-19-5954	FEMALE	TCGA-06-0414	MALE	TCGA-26-6173	MALE	TCGA-32-1987	FEMALE
TCGA-06-1805	FEMALE	TCGA-16-1063	MALE	TCGA-06-0125	FEMALE	TCGA-14-1450	FEMALE	TCGA-14-0736	MALE
TCGA-02-0058	FEMALE	TCGA-28-5204	MALE	TCGA-02-0085	FEMALE	TCGA-08-0246	FEMALE	TCGA-02-0290	MALE
TCGA-02-0080	MALE	TCGA-28-1749	MALE	TCGA-06-0184	MALE	TCGA-27-1830	MALE	TCGA-12-0819	FEMALE
TCGA-02-0104	FEMALE	TCGA-74-6575	FEMALE	TCGA-19-1786	FEMALE	TCGA-06-0156	MALE	TCGA-06-0132	MALE
TCGA-06-A7TL	FEMALE	TCGA-06-0649	FEMALE	TCGA-06-6695	MALE	TCGA-12-0828	MALE	TCGA-16-1045	FEMALE
TCGA-06-0882	MALE	TCGA-32-4210	MALE	TCGA-81-5910	MALE	TCGA-06-0397	FEMALE	TCGA-08-0357	MALE
TCGA-08-0355	FEMALE	TCGA-76-6660	MALE	TCGA-06-0177	MALE	TCGA-12-0620	MALE	TCGA-06-2564	MALE
TCGA-06-0129	MALE	TCGA-06-0158	MALE	TCGA-06-6388	FEMALE	TCGA-76-6280	MALE	TCGA-87-5896	FEMALE
TCGA-02-0069	FEMALE	TCGA-32-4719	MALE	TCGA-12-1089	MALE	TCGA-02-2470	MALE	TCGA-06-0749	MALE
TCGA-14-1821	MALE	TCGA-41-6646	FEMALE	TCGA-08-0531	MALE	TCGA-02-0289	MALE	TCGA-02-0003	MALE
TCGA-06-0221	MALE	TCGA-26-5134	MALE	TCGA-76-6285	FEMALE	TCGA-32-1978	MALE	TCGA-02-0285	FEMALE
TCGA-08-0245	FEMALE	TCGA-06-0878	MALE	TCGA-14-0790	FEMALE	TCGA-76-6191	MALE	TCGA-02-0422	MALE
TCGA-08-0350	MALE	TCGA-26-5132	MALE	TCGA-06-0133	MALE	TCGA-16-0848	MALE	TCGA-06-0881	MALE
TCGA-06-0151	FEMALE	TCGA-14-3476	MALE	TCGA-06-A7TK	MALE	TCGA-06-0240	MALE	TCGA-02-0064	MALE
TCGA-81-5911	MALE	TCGA-76-6192	MALE	TCGA-06-0179	MALE	TCGA-16-1062	FEMALE	TCGA-02-0015	MALE
TCGA-02-0027	FEMALE	TCGA-02-0037	FEMALE	TCGA-02-2486	MALE	TCGA-02-0068	MALE	TCGA-08-0358	MALE
TCGA-06-0146	FEMALE	TCGA-76-6657	MALE	TCGA-06-6693	FEMALE	TCGA-02-0079	MALE	TCGA-14-0786	FEMALE
TCGA-27-1836	FEMALE	TCGA-19-2631	FEMALE	TCGA-28-2502	MALE	TCGA-06-6699	FEMALE	TCGA-08-0347	MALE
TCGA-06-0176	MALE	TCGA-06-0149	FEMALE	TCGA-26-5139	FEMALE	TCGA-74-6578	MALE	TCGA-76-4932	FEMALE
TCGA-12-3653	FEMALE	TCGA-06-0159	MALE	TCGA-26-6174	FEMALE	TCGA-06-5856	MALE	TCGA-02-0016	MALE
TCGA-27-2521	MALE	TCGA-02-0321	MALE	TCGA-19-2623	MALE	TCGA-19-5953	MALE	TCGA-RR-A6KB	MALE
TCGA-08-0517	FEMALE	TCGA-08-0380	FEMALE	TCGA-06-6697	MALE	TCGA-06-6390	MALE	TCGA-74-6577	MALE
TCGA-02-0024	MALE	TCGA-08-0386	MALE	TCGA-32-1976	MALE	TCGA-12-1602	MALE	TCGA-19-2624	MALE

TCGA-16-1460	FEMALE	TCGA-12-0688	MALE	TCGA-14-0812	MALE	TCGA-12-1090	MALE	TCGA-06-0201	FEMALE
TCGA-19-4065	MALE	TCGA-14-0871	FEMALE	TCGA-06-2567	MALE	TCGA-08-0353	MALE	TCGA-19-1389	MALE
TCGA-26-1438	MALE	TCGA-06-5418	FEMALE	TCGA-06-0197	FEMALE	TCGA-06-0143	MALE	TCGA-06-0166	MALE
TCGA-15-1447	FEMALE	TCGA-12-0692	FEMALE	TCGA-12-0653	MALE	TCGA-19-1388	MALE	TCGA-14-1827	MALE
TCGA-14-0783	FEMALE	TCGA-12-1098	FEMALE	TCGA-06-0171	MALE	TCGA-76-4927	MALE	TCGA-06-5411	MALE
TCGA-02-0084	FEMALE	TCGA-06-1087	MALE	TCGA-15-1449	MALE	TCGA-14-1823	FEMALE	TCGA-19-1386	MALE
TCGA-27-1837	MALE	TCGA-08-0510	MALE	TCGA-15-0742	MALE	TCGA-32-2494	FEMALE	TCGA-06-0394	MALE
TCGA-12-0616	FEMALE	TCGA-12-0775	FEMALE	TCGA-12-1095	FEMALE	TCGA-12-1092	MALE	TCGA-08-0375	FEMALE
TCGA-02-0258	FEMALE	TCGA-41-2575	MALE	TCGA-06-0241	FEMALE	TCGA-14-1402	FEMALE	TCGA-12-0780	FEMALE
TCGA-02-0432	MALE	TCGA-06-2558	FEMALE	TCGA-28-5209	FEMALE	TCGA-76-6662	MALE	TCGA-02-0330	FEMALE
TCGA-06-0194	FEMALE	TCGA-06-0237	FEMALE	TCGA-14-1795	MALE	TCGA-06-0192	MALE	TCGA-06-0147	FEMALE
TCGA-28-2512	MALE	TCGA-12-1598	FEMALE	TCGA-76-4934	FEMALE	TCGA-41-2573	MALE	TCGA-12-0662	MALE
TCGA-02-0114	FEMALE	TCGA-12-0829	MALE	TCGA-16-1047	FEMALE	TCGA-26-5133	MALE	TCGA-02-0116	MALE
TCGA-14-3477	FEMALE	TCGA-06-6700	MALE	TCGA-76-6656	MALE	TCGA-14-1794	MALE	TCGA-28-5216	MALE
TCGA-02-0023	FEMALE	TCGA-28-1745	MALE	TCGA-02-0060	FEMALE	TCGA-12-5301	MALE	TCGA-14-1395	MALE
TCGA-12-0826	FEMALE	TCGA-06-2557	MALE	TCGA-06-0214	MALE	TCGA-08-0359	FEMALE	TCGA-28-1755	FEMALE
TCGA-12-0827	FEMALE	TCGA-76-4929	FEMALE	TCGA-12-1093	FEMALE	TCGA-06-0745	MALE	TCGA-06-0208	FEMALE
TCGA-06-0178	MALE	TCGA-06-0182	MALE	TCGA-27-1831	MALE	TCGA-27-1832	FEMALE	TCGA-12-0776	MALE
TCGA-19-A60I	MALE	TCGA-19-2625	FEMALE	TCGA-32-5222	MALE	TCGA-02-0004	MALE	TCGA-06-0165	MALE
TCGA-19-1788	MALE	TCGA-32-1982	FEMALE	TCGA-14-1037	FEMALE	TCGA-27-1838	FEMALE	TCGA-12-0769	MALE
TCGA-14-1451	MALE	TCGA-06-0410	FEMALE	TCGA-02-0057	FEMALE	TCGA-32-2495	FEMALE	TCGA-02-0115	MALE
TCGA-06-0650	FEMALE	TCGA-76-4925	MALE	TCGA-06-0128	MALE	TCGA-41-5651	FEMALE	TCGA-08-0525	MALE
TCGA-02-0028	MALE	TCGA-06-6694	FEMALE	TCGA-06-0744	MALE	TCGA-08-0389	MALE	TCGA-16-0850	FEMALE
TCGA-14-4157	MALE	TCGA-06-0209	MALE	TCGA-08-0344	MALE	TCGA-32-1970	MALE	TCGA-02-0089	MALE
TCGA-28-1750	FEMALE	TCGA-19-5951	FEMALE	TCGA-06-5413	MALE	TCGA-06-2565	MALE	TCGA-19-5950	FEMALE
TCGA-19-A6J5	MALE	TCGA-06-0148	MALE	TCGA-06-0219	MALE	TCGA-06-0168	FEMALE	TCGA-14-2554	FEMALE
TCGA-06-0206	MALE	TCGA-12-3649	MALE	TCGA-14-0867	MALE	TCGA-02-0083	FEMALE	TCGA-28-5208	MALE
TCGA-06-0139	MALE	TCGA-08-0360	MALE	TCGA-02-0456	FEMALE	TCGA-08-0356	FEMALE	TCGA-08-0354	FEMALE
TCGA-02-0317	MALE	TCGA-28-1746	FEMALE	TCGA-74-6573	MALE	TCGA-12-3646	FEMALE	TCGA-27-2518	MALE
TCGA-12-0820	MALE	TCGA-28-2509	FEMALE	TCGA-06-0127	MALE	TCGA-06-6701	MALE	TCGA-76-4935	FEMALE
TCGA-32-4209	MALE	TCGA-74-6581	MALE	TCGA-02-0430	FEMALE	TCGA-26-1443	FEMALE	TCGA-06-0879	MALE
TCGA-02-0007	FEMALE	TCGA-19-0962	FEMALE	TCGA-02-0339	MALE	TCGA-06-5415	MALE	TCGA-28-1753	MALE
TCGA-08-0351	MALE	TCGA-06-0413	FEMALE	TCGA-28-5220	MALE	TCGA-08-0392	MALE	TCGA-06-6698	FEMALE
TCGA-02-0338	MALE	TCGA-02-0333	FEMALE	TCGA-41-2572	MALE	TCGA-14-0862	MALE	TCGA-02-2485	MALE
TCGA-28-1752	FEMALE	TCGA-06-0648	MALE	TCGA-14-0865	MALE	TCGA-28-6450	MALE	TCGA-06-0145	FEMALE
TCGA-28-5211	MALE	TCGA-12-3651	MALE	TCGA-06-0124	MALE	TCGA-06-0646	MALE	TCGA-06-A6S1	FEMALE
TCGA-06-1086	MALE	TCGA-19-5959	FEMALE	TCGA-27-1833	FEMALE	TCGA-14-1452	MALE	TCGA-06-0747	MALE
TCGA-02-0102	MALE	TCGA-28-1756	MALE	TCGA-32-2638	MALE	TCGA-02-0034	MALE	TCGA-02-0071	MALE
TCGA-02-2483	MALE	TCGA-06-0877	MALE	TCGA-19-4068	FEMALE	TCGA-12-5295	FEMALE	TCGA-06-0686	MALE
TCGA-26-1442	MALE	TCGA-14-1396	FEMALE	TCGA-06-0169	MALE	TCGA-14-1034	FEMALE	TCGA-12-0778	MALE
TCGA-02-0113	FEMALE	TCGA-14-0813	MALE	TCGA-19-A6J4	MALE	TCGA-32-1991	MALE	TCGA-06-2561	FEMALE
TCGA-06-0750	MALE	TCGA-06-A5U1	FEMALE	TCGA-12-1099	FEMALE	TCGA-08-0518	FEMALE	TCGA-27-1835	FEMALE
TCGA-02-0051	MALE	TCGA-76-6193	MALE	TCGA-76-4926	MALE	TCGA-76-6286	MALE	TCGA-19-5956	FEMALE
TCGA-06-0138	MALE	TCGA-02-0281	FEMALE	TCGA-OX-A56R	MALE	TCGA-19-2629	MALE	TCGA-28-5214	MALE
TCGA-06-0409	MALE	TCGA-06-5412	FEMALE	TCGA-26-1799	MALE	TCGA-12-0670	MALE	TCGA-12-1088	FEMALE

TCGA-02-0021	FEMALE	TCGA-02-0047	MALE	TCGA-02-0059	FEMALE	TCGA-12-0619	MALE	TCGA-4W-AA9T	FEMALE
TCGA-14-2555	FEMALE	TCGA-12-0615	FEMALE	TCGA-02-0074	FEMALE	TCGA-12-3652	MALE	TCGA-16-0849	MALE
TCGA-06-6391	FEMALE	TCGA-26-5136	FEMALE	TCGA-02-0269	MALE	TCGA-4W-AA9R	MALE	TCGA-06-0185	MALE
TCGA-28-1747	MALE	TCGA-08-0352	MALE	TCGA-06-0152	MALE	TCGA-28-1751	FEMALE	TCGA-02-0033	MALE
TCGA-02-0054	FEMALE	TCGA-27-2526	FEMALE	TCGA-32-1986	MALE	TCGA-06-5414	MALE	TCGA-06-0174	MALE
TCGA-06-0167	MALE	TCGA-06-A6S0	MALE	TCGA-4W-AA9S	MALE	TCGA-06-0875	FEMALE	TCGA-14-1401	MALE
TCGA-02-0001	FEMALE	TCGA-06-0939	FEMALE	TCGA-28-2513	FEMALE	TCGA-14-1043	MALE	TCGA-14-1458	MALE
TCGA-76-6663	FEMALE	TCGA-02-0048	MALE	TCGA-12-0657	MALE	TCGA-14-1455	MALE	TCGA-02-0039	MALE
TCGA-06-5417	FEMALE	TCGA-32-2632	MALE	TCGA-14-1453	MALE	TCGA-02-0046	MALE	TCGA-14-0789	MALE
TCGA-28-2514	MALE	TCGA-19-1387	MALE	TCGA-14-0787	MALE	TCGA-02-0446	MALE	TCGA-02-0106	MALE
TCGA-06-5858	FEMALE	TCGA-06-0142	MALE	TCGA-19-1789	FEMALE	TCGA-06-0155	MALE	TCGA-06-5408	FEMALE
TCGA-02-0086	FEMALE	TCGA-06-0119	FEMALE	TCGA-06-0175	MALE	TCGA-02-0009	FEMALE	TCGA-06-0130	MALE
TCGA-06-0150	MALE	TCGA-41-3393	FEMALE	TCGA-08-0373	MALE	TCGA-02-0325	MALE	TCGA-06-0154	MALE
TCGA-12-0818	FEMALE	TCGA-06-2562	MALE	TCGA-14-0817	FEMALE	TCGA-19-0963	MALE	TCGA-02-0260	MALE
TCGA-02-0099	MALE	TCGA-06-1804	FEMALE	TCGA-02-0324	FEMALE	TCGA-12-1097	MALE	TCGA-02-0043	FEMALE
TCGA-08-0349	MALE	TCGA-27-2527	MALE	TCGA-08-0511	MALE	TCGA-06-1802	MALE	TCGA-76-6661	MALE
TCGA-12-3650	MALE	TCGA-19-1791	FEMALE	TCGA-08-0346	MALE	TCGA-02-2466	MALE	TCGA-06-1084	MALE
TCGA-06-0238	MALE	TCGA-32-2634	MALE	TCGA-19-1385	MALE	TCGA-08-0522	MALE	TCGA-14-1454	FEMALE
TCGA-12-0703	MALE	TCGA-02-0326	FEMALE	TCGA-08-0514	FEMALE	TCGA-06-0216	FEMALE	TCGA-74-6584	FEMALE
TCGA-12-0822	MALE	TCGA-19-0960	FEMALE	TCGA-08-0390	MALE	TCGA-06-1800	MALE	TCGA-19-2619	FEMALE
TCGA-02-0332	FEMALE	TCGA-19-2621	MALE	TCGA-32-1979	FEMALE	TCGA-12-3648	FEMALE	TCGA-15-1446	MALE
TCGA-12-0772	MALE	TCGA-19-5955	MALE	TCGA-06-0743	MALE	TCGA-02-0055	FEMALE	TCGA-06-0213	FEMALE
TCGA-26-A7UX	MALE	TCGA-06-2559	MALE	TCGA-06-0187	MALE	TCGA-06-0141	MALE	TCGA-06-0645	FEMALE
TCGA-32-4213	FEMALE	TCGA-06-0122	FEMALE	TCGA-19-0964	MALE	TCGA-06-0190	MALE	TCGA-12-1096	MALE
TCGA-28-5219	FEMALE	TCGA-76-4928	FEMALE	TCGA-02-0070	MALE	TCGA-12-0821	MALE	TCGA-16-1056	MALE
TCGA-06-0162	FEMALE	TCGA-16-0846	MALE	TCGA-02-0439	FEMALE	TCGA-28-5215	FEMALE		
TCGA-19-5947	FEMALE	TCGA-06-0140	MALE	TCGA-19-2620	MALE	TCGA-02-0440	MALE		
TCGA-06-0211	MALE	TCGA-06-0126	MALE	TCGA-14-1825	MALE	TCGA-27-2528	MALE		
TCGA-06-1806	MALE	TCGA-12-1600	MALE	TCGA-16-1060	FEMALE	TCGA-32-2615	MALE		
TCGA-12-1599	FEMALE	TCGA-41-3392	MALE	TCGA-76-4931	FEMALE	TCGA-02-0451	FEMALE		
TCGA-02-0025	MALE	TCGA-41-2571	MALE	TCGA-08-0520	MALE	TCGA-19-5952	MALE		

Supplementary Table 1. The gender of the individuals in the study

This table presents a detailed account of the gender distribution among the participants involved in the study.

Gene	HR	HR.95L	HR.95H	pValue
AC008708.2	1.234127	1.000332	1.522564	0.049639
Z98257.1	1.254375	1.010806	1.556636	0.039634
AC009948.2	1.179578	1.024162	1.358578	0.021953
DLEU1	0.793696	0.669591	0.940802	0.007736
DBH-AS1	1.068982	1.003568	1.138659	0.038405
AC005632.5	3.331854	1.738213	6.386589	0.000289
LINC01018	1.132318	1.011489	1.26758	0.030899
ZMIZ1-AS1	1.532644	1.26711	1.853823	1.09E-05
AC019254.1	1.484502	1.059542	2.079906	0.021671
AC106738.1	1.23055	1.095948	1.381684	0.000448
AC108673.2	1.214409	1.045586	1.410491	0.010969
FAM153CP	2.222743	1.158218	4.265681	0.016324
CD2BP2-DT	1.06934	1.00307	1.139989	0.039989
AC099684.1	1.699202	1.020082	2.830445	0.041717
AC147067.1	1.169628	1.064871	1.28469	0.001065
AC126407.1	0.948475	0.904769	0.994292	0.027964
AC132938.3	1.059043	1.00955	1.110962	0.018813
AC002456.1	1.016526	1.002043	1.031217	0.025172
ITGA6-AS1	1.423342	1.137466	1.781066	0.002029
HOTAIRM1	1.008839	1.001017	1.016722	0.02669
AC004847.1	1.332498	1.13371	1.566143	0.000497
AC010226.1	1.060392	1.002928	1.12115	0.039131
AC009271.1	0.826361	0.68991	0.989798	0.038328
OBSCN-AS1	1.389731	1.018674	1.895947	0.037828
AC012615.1	0.986552	0.974095	0.999169	0.036776
AC104971.1	1.170763	1.013777	1.352058	0.031854
LINC01993	1.213199	1.024724	1.436341	0.024866
AC243829.1	1.967933	1.261795	3.069247	0.002832
AC124248.1	1.105661	1.020617	1.197792	0.013905
LINC02598	0.905373	0.829484	0.988205	0.02604
AL035530.2	0.869162	0.757802	0.996887	0.045014
ELN-AS1	1.038178	1.011239	1.065836	0.005221
LINC02773	1.128962	1.006461	1.266373	0.038465
AP000695.2	1.537242	1.175412	2.010456	0.001688
VIM-AS1	1.304175	1.065904	1.59571	0.00988
AC025171.1	1.058707	1.019495	1.099428	0.00305
AP001330.4	1.142711	1.01942	1.280913	0.022012
AC078883.1	1.621752	1.238551	2.123512	0.000439
MIR3945HG	1.220056	1.080576	1.377539	0.001322
AP005329.1	1.232159	1.060495	1.431611	0.006386
LINC02328	1.067723	1.00137	1.138472	0.045307
CASC8	1.467012	1.056736	2.036576	0.02204
AL365184.1	1.940806	1.005432	3.746378	0.048143
AC011405.1	0.187138	0.055205	0.634371	0.007131

AC104109.2	1.181417	1.044074	1.336826	0.008194
AL391832.2	1.580571	1.015239	2.460705	0.04267
AC093627.1	1.383712	1.125296	1.70147	0.002076
SLC25A21-AS1	0.909508	0.831345	0.99502	0.038559
CATIP-AS1	1.7712	1.171778	2.677255	0.006688
DCST1-AS1	1.056571	1.002948	1.113061	0.038384
APCDD1L-DT	1.120515	1.03538	1.21265	0.004767
AC068643.1	0.92286	0.851876	0.999758	0.049311
AC083805.2	1.027607	1.005387	1.050319	0.01462
LINC00460	1.041213	1.00314	1.08073	0.033594
AC091182.2	1.125861	1.045451	1.212456	0.001715
SNHG1	0.992673	0.985858	0.999535	0.036424
AC090125.1	1.186085	1.029173	1.36692	0.018416
AC018645.3	1.01737	1.001413	1.033582	0.032756
AC010655.2	1.045761	1.00284	1.090518	0.036384
LINC00426	2.453556	1.146757	5.249533	0.020732
PRKAG2-AS1	1.020952	1.000941	1.041362	0.040059
HCG21	1.151437	1.024087	1.294623	0.018375
AC138696.2	1.039164	1.002916	1.076722	0.033945
AC245128.3	1.074043	1.013895	1.137759	0.015129
LINC02761	0.771917	0.599598	0.993759	0.044586
AC138207.4	1.066485	1.007192	1.129269	0.027419
AC131009.1	1.35041	1.170152	1.558437	3.96E-05
C2CD4D-AS1	1.042508	1.00931	1.076798	0.011695
AGAP2-AS1	1.001863	1.000704	1.003023	0.001619
AC008035.1	1.007292	1.003121	1.01148	0.000599
AC084117.1	1.112876	1.011214	1.224759	0.028661
LINC01503	1.01975	1.00829	1.03134	0.000695
LINC01605	1.064816	1.024221	1.107019	0.001542
C8orf49	1.39898	1.021794	1.915401	0.036219
LINC01852	1.170899	1.00698	1.361502	0.04033
SOX21-AS1	0.96561	0.942739	0.989036	0.004217
TMEM220-AS1	1.059748	1.003597	1.119041	0.036689
AC008567.2	0.633876	0.411381	0.976708	0.038752
AC015908.3	1.078008	1.014868	1.145076	0.014719
AP000880.1	1.109756	1.001981	1.229122	0.045723
PARAL1	1.842929	1.173545	2.894128	0.007933
AC005225.1	2.23155	1.028648	4.841125	0.04221
AC092171.2	1.019787	1.001821	1.038074	0.030725
ZNF197-AS1	0.884293	0.800184	0.977243	0.015892
AL049836.1	1.17387	1.073211	1.28397	0.000457
AC022784.5	1.448936	1.107397	1.895811	0.006856
AC074286.1	0.754998	0.572305	0.996011	0.046785
AC021594.1	1.631156	1.234091	2.155976	0.000586
AL606760.1	1.12416	1.023934	1.234197	0.014035

AC105046.1	4.248178	1.392085	12.96402	0.011051
ZEB1-AS1	0.897638	0.832007	0.968447	0.00531
OSMR-AS1	1.211545	1.07366	1.367139	0.001853
POLR2J4	1.069132	1.011564	1.129976	0.017928
MANCR	1.681145	1.035467	2.729443	0.035648
AC010504.1	1.15341	1.001545	1.328302	0.047548
AC090796.1	1.084305	1.000925	1.17463	0.04741
CYP4A22-AS1	1.541197	1.059202	2.242526	0.023788
ZKSCAN7-AS1	0.895113	0.815321	0.982713	0.020018
BNC2-AS1	1.049492	1.007447	1.093292	0.02058
AC068888.1	1.099531	1.02656	1.177689	0.006766
AC107959.3	1.720486	1.106507	2.675151	0.01598
AC122108.2	0.417789	0.182219	0.957902	0.03925
AC026367.2	1.06002	1.006001	1.11694	0.028949
AC144831.1	1.134867	1.014222	1.269863	0.027368
MBNL1-AS1	0.754572	0.575731	0.988966	0.041315
AL391056.1	2.071375	1.215951	3.528591	0.007376
AC004264.1	1.169851	1.022333	1.338654	0.02254
LINC01992	2.25443	1.098799	4.625465	0.026629
HOXC13-AS	1.039542	1.003866	1.076486	0.029514
AL353593.1	1.925795	1.020982	3.632469	0.04296
AC013391.3	0.978518	0.962226	0.995086	0.011245
AC010624.2	1.082711	1.001859	1.170087	0.044763
DLGAP1-AS4	1.066401	1.002031	1.134905	0.042986
AC079298.3	1.391768	1.156309	1.675174	0.000473
LINC02036	1.204638	1.029882	1.409048	0.019903
SNHG18	1.018047	1.001071	1.03531	0.037098
AL138999.1	1.639038	1.051348	2.555242	0.029185
LINC02857	1.501543	1.064617	2.117786	0.020512
AL356215.1	1.083526	1.004341	1.168955	0.038281
LINC01614	1.020816	1.002163	1.039816	0.028552
DGUOK-AS1	1.070308	1.018856	1.124359	0.006869
ZBTB44-DT	2.134945	1.23266	3.697687	0.006802
MIR497HG	1.029702	1.003648	1.056432	0.025189
PRKCQ-AS1	0.903075	0.825581	0.987843	0.025936
AL022344.1	0.391338	0.205185	0.746378	0.0044
AC239800.2	0.866782	0.760639	0.987737	0.031945
AC005264.1	5.582796	2.420409	12.877	5.51E-05
LINC00968	1.156936	1.012558	1.321901	0.032076
AC066612.1	3.001072	1.115757	8.072041	0.029485
SYP-AS1	1.786675	1.036308	3.080365	0.036771
LRRC8C-DT	0.862467	0.777932	0.956188	0.004936
AL139385.1	4.164893	1.8789	9.232174	0.000443
AC004816.1	1.060609	1.002656	1.121911	0.040124
AL357033.4	1.152739	1.009336	1.316517	0.035988

AC140125.2	1.940525	1.265233	2.976241	0.002381
AC072061.1	1.12626	1.005823	1.261118	0.039343
AC011899.2	1.215233	1.075792	1.372748	0.00172
Z99774.1	7.832616	1.113673	55.08785	0.038627
AC067969.1	1.398944	1.076391	1.818153	0.012058
CERNA1	1.252039	1.035118	1.514417	0.020584
AL137786.1	1.448805	1.163512	1.804052	0.000921
AC080013.1	1.524247	1.128393	2.058972	0.006009
AC145343.1	1.197179	1.05449	1.359177	0.005447
AC008738.4	1.06973	1.007583	1.135711	0.02729
AL161629.1	0.800441	0.664589	0.964064	0.018995
TMEM26-AS1	2.787942	1.564145	4.969247	0.000507
DPP10-AS1	0.944631	0.894327	0.997766	0.041342
LINC01936	1.378726	1.09694	1.732898	0.005903
AC100812.1	1.154172	1.053052	1.265002	0.002177
AC093388.1	1.141628	1.017122	1.281376	0.02457
AC106820.3	1.030445	1.001225	1.060518	0.041013
AL354919.2	1.009614	1.001768	1.017522	0.016231
GLIDR	0.882546	0.798459	0.975488	0.014455
SOX1-OT	1.015105	1.002687	1.027677	0.016973
LINC02192	1.485798	1.056545	2.089446	0.022836
LINC01034	2.565537	1.393563	4.723132	0.00248
ECE1-AS1	2.029475	1.148794	3.5853	0.01478
AL356414.1	1.42229	1.086467	1.861914	0.010364
LINC01127	1.377562	1.134006	1.673428	0.001252
ZBTB11-AS1	1.189031	1.019399	1.386889	0.027481
LEF1-AS1	1.202733	1.041431	1.389018	0.011988
AL118508.1	2.045716	1.13296	3.693821	0.017596
AC116456.1	2.132339	1.024627	4.437584	0.042865
AC066612.2	1.84063	1.147121	2.953411	0.011443
AL355922.1	1.019685	1.001635	1.038061	0.032418
PLBD1-AS1	1.061141	1.003776	1.121785	0.03636
MYOSLID	1.099483	1.0423	1.159802	0.000501
MIR181A2HG	0.957667	0.920417	0.996425	0.032609
AC087045.2	1.99917	1.251551	3.193384	0.003744
LINC01929	1.886268	1.091374	3.260117	0.023016
MIR7-3HG	1.034649	1.004592	1.065605	0.023538
MIR155HG	1.042779	1.005464	1.081478	0.024255
PDE2A-AS2	1.20978	1.029574	1.421528	0.020661
AL133297.1	1.1015	1.011129	1.199948	0.026873
AL353796.1	0.818516	0.699795	0.957379	0.012255
SNHG29	0.998749	0.997564	0.999936	0.038827
LINC01943	1.289021	1.132394	1.467312	0.000123
ZNF22-AS1	0.922188	0.864196	0.984071	0.014505
AC073370.1	1.803027	1.080906	3.007573	0.023947

LINC02384	1.329852	1.057632	1.672137	0.014709
AL132800.1	0.808297	0.670515	0.974392	0.025613
SOCAR	1.439683	1.120841	1.849223	0.004329
AC026367.1	1.02261	1.002986	1.042619	0.023725
AC061992.2	1.057349	1.016498	1.099841	0.005538
AP006333.2	0.891834	0.804431	0.988734	0.02961
AC068057.2	1.180747	1.003552	1.389228	0.045209
LINC02458	1.691095	1.04958	2.724711	0.030865
LINC00158	0.51492	0.294778	0.899464	0.019686
AC011603.2	1.838602	1.108683	3.049077	0.018288
BX640514.2	1.073848	1.016684	1.134228	0.010686
AC092287.1	1.395544	1.101206	1.768554	0.005822
AL135905.1	1.580399	1.123454	2.223196	0.008576
AC103858.1	2.233033	1.116276	4.467028	0.023153
AC073115.2	1.551116	1.063183	2.262979	0.022733
HAR1B	1.108284	1.008889	1.217473	0.031989
AL157394.1	1.289257	1.040675	1.597216	0.020083
AC010327.4	2.266544	1.228357	4.182188	0.008844
CYP1B1-AS1	1.155376	1.00105	1.333492	0.048346
SAPCD1-AS1	1.607077	1.052197	2.454575	0.028134
MIR210HG	1.01747	1.001039	1.03417	0.037068
LINC01574	1.36776	1.165152	1.605599	0.000129
AC007384.1	1.392267	1.024882	1.891348	0.034243
AL133215.2	0.860611	0.747928	0.99027	0.036035
PPP1R14B-AS1	1.044038	1.016047	1.0728	0.001883
LINC00957	1.035228	1.00713	1.06411	0.013663
AC010457.1	1.092034	1.001923	1.190249	0.045103
CYTOR	1.011231	1.002198	1.020347	0.014709
AC027644.3	1.044737	1.014927	1.075422	0.003045
LINC01952	1.076362	1.004096	1.15383	0.037965
LINC01465	1.226615	1.062092	1.416624	0.005439
AC018413.1	0.689966	0.503966	0.944612	0.020586
AC025171.3	1.144046	1.012833	1.292259	0.030379
AC021613.1	1.084826	1.014353	1.160194	0.017511
LINC00886	1.100976	1.014415	1.194925	0.021306
AC096921.2	1.610345	1.088442	2.382496	0.017125
AC002070.1	1.051733	1.012379	1.092617	0.009534
AL512625.1	0.879153	0.773042	0.999829	0.049696
AC004817.3	1.192494	1.070214	1.328747	0.001426
AC010654.1	1.206396	1.05233	1.383018	0.00711
AC136475.3	1.450821	1.120605	1.878344	0.004741
LINC02320	1.248087	1.094134	1.423702	0.000969

Supplementary Table 2. Results of univariate Cox regression analysis of the entire cohort.

Results of univariate Cox regression analysis showing p-values and hazard ratios (HR) with confidence intervals (CI) for 221 differentially expressed genes across the cohort.

ID	Coef	HR	HR.95L	HR.95H	pValue
AC011405.1	-2.68009	0.068557	0.014887	0.315726	0.000583
AGAP2-AS1	0.130556	1.139462	0.980575	1.324095	0.088391
LINC01503	0.251445	1.285883	0.94669	1.746605	0.107543
HOXC13-AS	0.201458	1.223184	1.022264	1.463594	0.027772
AL139385.1	1.101564	3.008869	0.898528	10.07569	0.074028
LINC01127	0.637009	1.890816	1.229178	2.908598	0.003743
LINC01574	0.772119	2.164347	1.434325	3.265926	0.000235

Supplementary Table 3. Results of multivariate Cox regression.

The results showed that the p-values and hazard ratios of the 4 differentially expressed Graphene Therapy-related LncRNAs ($p<0.05$) were finalized with confidence intervals.
